# External validation of QUiPP App in three independent European cohorts of symptomatic women

**DOI:** 10.1002/uog.29263

**Published:** 2025-06-26

**Authors:** A. M. Fischer, K. Bos, P. C. A. M. Bakker, M. Hoogendoorn, B. W. Mol, A. L. Rietveld, P. W. Teunissen, I. Dehaene, F. Hermans

**Affiliations:** ^1^ Department of Obstetrics and Gynecology Amsterdam UMC location AMC Amsterdam The Netherlands; ^2^ Amsterdam Reproduction and Development Amsterdam The Netherlands; ^3^ Department of Computer Science Vrije Universiteit Amsterdam The Netherlands; ^4^ Department of Obstetrics and Gynecology Monash University Melbourne Victoria Australia; ^5^ Aberdeen Centre for Women's Health Research University of Aberdeen Aberdeen UK; ^6^ Department of Gynecology and Obstetrics Maastricht UMC Maastricht The Netherlands; ^7^ School of Health Professions Education, Faculty of Health Medicine and Life Sciences Maastricht University Maastricht The Netherlands; ^8^ Department of Obstetrics and Gynecology Ghent University Hospital Ghent Belgium

**Keywords:** prediction, risk assessment, spontaneous preterm birth, validation

## Abstract

**Objective:**

To validate externally the QUantitative Innovation in Predicting Preterm birth (QUiPP) App v.2 for the prediction of spontaneous preterm birth (sPTB) in symptomatic women attending tertiary care in Europe.

**Methods:**

The QUiPP App v.2 was validated in three independent datasets: a prospective European multicenter cohort across five countries (*n* = 452), a retrospective single‐center cohort in The Netherlands (*n* = 581) and a retrospective single‐center cohort in Belgium (*n* = 399). The cohorts consisted of pregnant women between 23 and 34 weeks' gestation with symptoms of threatened preterm birth attending a tertiary care hospital between 2012 and 2023. We calculated risk estimates using the QUiPP App v.2 by inputting quantitative fetal fibronectin (qfFN) and/or cervical length (CL) measurement, in addition to other risk factors. As a result of the absence of a fibronectin detection kit in the Belgian cohort, only the QUiPP model based on CL could be validated in this dataset. The European cohort had no missing cases, but for the Dutch cohort, only complete cases were analyzed due to missing data. For the Belgian cohort, we statistically corrected for patients lost to follow‐up using inverse probability of censoring weighting. Discrimination was assessed using receiver‐operating‐characteristics (ROC)‐curve analysis of three QUiPP models (qfFN alone, CL alone and CL plus qfFN) for the risk of sPTB at six predefined timepoints: within 1, 2 and 4 weeks after testing, and at < 30, < 34 and < 37 weeks' gestation. Sensitivity, specificity and positive and negative likelihood ratios were calculated using risk thresholds of 5%, 10% and 15%. Model calibration was assessed to evaluate the agreement between expected and observed outcomes.

**Results:**

The predictive performance of the QUiPP App v.2 for sPTB within 1 week after testing had an area under the ROC curve (AUC) of 0.84 (95% CI, 0.79–0.89) and 0.74 (95% CI, 0.66–0.83) in the European and Dutch cohorts, respectively, using the combined model of CL plus qfFN, and 0.80 (95% CI, 0.75–0.85) in the Belgian cohort using the CL‐only model. Predictive performance was greater for shorter‐term outcomes, specifically sPTB < 30 weeks, compared with longer‐term outcomes, such as sPTB < 37 weeks. The highest AUC (0.91 (95% CI, 0.86–0.95)) was achieved by the model using CL plus qfFN for the prediction of sPTB < 30 weeks in the European cohort. Calibration was excellent for patients with negligible risk; however, for women at greater risk of sPTB, the risk was generally underestimated compared with the observed event rate.

**Conclusions:**

The QUiPP App v.2 offers reassurance for patients with low predicted risk of sPTB and has greater predictive performance for shorter‐term, compared with longer‐term, outcomes. Despite significant differences in prevalence from the original QUiPP dataset, the model combining CL plus qfFN and the qfFN‐only model perform reasonably well. Statistical correction for patients lost to follow‐up in the dataset comprising censored and uncensored patients improved the discriminative ability of the CL predictor. © 2025 The Author(s). *Ultrasound in Obstetrics & Gynecology* published by John Wiley & Sons Ltd on behalf of International Society of Ultrasound in Obstetrics and Gynecology.

## INTRODUCTION

In Europe, the rate of preterm birth (PTB) varies between 5% and 10% among live births^1^, ^2^. PTB contributes significantly to neonatal morbidity and mortality, with lifelong effects in survivors^3^, ^4^. PTB remains difficult to predict, even in women who exhibit symptoms suggestive of threatened PTB^5^. Both under‐ and overtreatment of threatened PTB can have negative consequences^4^, ^6^.

Hospital admissions for threatened PTB represent a burden for obstetric departments and are accompanied by high healthcare costs and use of scarce resources^6^, ^7^. New strategies are warranted to improve the identification of women in need of treatment, such as hospital admission, tocolysis and/or corticosteroids for fetal lung maturation. Validated risk prediction models are one such prognostic strategy that can aid clinical decision‐making. The strongest predictors for spontaneous PTB (sPTB) are shortened cervical length (CL) and increased fetal fibronectin (fFN)^8^, ^9^, ^10^, ^11^, ^12^. However, because of the relatively low incidence of sPTB, both CL measurement and the quantitative fFN (qfFN) test are characterized by a high negative predictive value but poor positive predictive value, leading to overtreatment of women with a false‐positive test result. Since 2020, an updated version (v.2) of the risk prediction tool called the QUantitative Innovation in Predicting Preterm birth (QUiPP™) App has been available for assessing the risk of sPTB in symptomatic women with regular contractions and intact membranes^13^, ^14^. This application calculates a risk estimate for sPTB at different timepoints based on a combination of CL, qfFN and maternal risk factors.

However, the QUiPP App v.2 was developed and validated using only data from the UK, and its performance when extrapolated to other countries with different diagnostic and treatment policies is yet to be assessed. Additionally, it is necessary to assess the calibration of the model to identify any systematic over‐ or underestimation of risk for patients in various contexts. This is important because of significant variation in the prevalence of PTB across different populations, even within Europe^2^.

At the time of writing, the QUiPP App v.2 has not yet been validated externally within the symptomatic population. Therefore, the aim of this study was to validate externally the QUiPP App v.2 using three different populations to assess its predictive performance for clinical decision‐making.

## METHODS

### QUiPP App study

The QUiPP App v.2^13^ has three models running in the background: one based on CL alone, one based on qfFN alone and one based on both CL and qfFN. In addition to these variables, the following risk factors are included in each model: previous cervical surgery, previous sPTB < 37 weeks' gestation, previous preterm prelabor rupture of membranes (PPROM) < 37 weeks' gestation, number of fetuses and gestational age at the time of CL measurement and/or qfFN test. All models are based on a log‐normal survival curve and provide risk estimates for sPTB at < 30, < 34 and < 37 weeks' gestation and within 1, 2 and 4 weeks after testing. The survival models were constructed by designating patients with sPTB occurring before 37 weeks' gestation as events, while censoring patients for whom delivery was at or after 37 weeks. Censoring in this context refers to patients who were event‐free (i.e. no sPTB) at 37 weeks' gestation. Iatrogenic PTB was considered a non‐event and was censored at 37 weeks. In instances in which PPROM preceded iatrogenic PTB, it was classified as spontaneous PTB. In twin pregnancy, gestational age at delivery of the firstborn twin was used in the analysis^13^.

The QUiPP App can be used for pregnant women between 23 + 0 and 34 + 6 weeks' gestation with symptoms of threatened PTB, such as abdominal pain or tightening. Women receiving an intervention intended to reduce the risk of sPTB (e.g. tocolysis, cerclage) were included in the analysis^13^. Exclusion criteria were: established labor, ruptured membranes, significant antepartum hemorrhage, sexual intercourse within the past 24 h, major fetal abnormality and triplets or higher‐order multiple pregnancy.

The current version (v.2) of the QUiPP App for symptomatic women was launched in 2020^13^. The model development dataset comprised 1173 observations from 1032 women with qfFN test results and 229 observations from 204 women with both qfFN and CL measurements. Observations from women with both qfFN and CL measurements were also used to develop models using just one of the measurements as predictor. Data were collected between October 2010 and October 2017 from 16 hospitals in the UK. The development dataset consisted predominantly of women with European ethnicity (54.9%), followed by women with Asian ethnicity (27.1%). Mean ± SD maternal age was 29.9 ± 5.7 years and mean body mass index was 26.1 ± 5.9 kg/m^2^. Furthermore, 15.3% of patients had experienced previous PTB < 37 weeks, 6.3% had a history of cervical surgery and 4.0% were twin pregnancies.

The validation dataset was gathered between June 2017 and March 2018 and comprised 588 observations from 506 women: 576 qfFN test results from 502 women, 155 CL measurements from 132 women, and 143 observations with both qfFN and CL measurements from 128 women^13^. The areas under the receiver‐operating‐characteristics (ROC) curves (AUCs) in the validation set for the prediction of sPTB at different timepoints were fairly high, varying between 0.77 (sPTB < 37 weeks' gestation) and 0.96 (sPTB < 30 weeks' gestation). Sensitivity and specificity were calculated in a similar manner to that in the QUiPP study^13^, using a risk threshold of 5% to indicate a positive test.

### External validation

#### Datasets

For this external validation study, three datasets were used. The first dataset was a secondary analysis derived from a prospective trial, whereas the second dataset was collected retrospectively, containing only patients with a high‐risk PTB profile for whom the outcome was known. The third dataset was also collected retrospectively and comprised patients hospitalized for threatened PTB with either complete or incomplete outcome information. Due to these inhomogeneous characteristics, the datasets were analyzed separately. The advantage of this heterogeneity is that it enables evaluation of the influence of patient selection on model performance and better reflects real‐world clinical practice.

#### European Fibronectin Study

The first dataset is from the European Fibronectin Study (EUFIS), a European multicenter cohort study published by Bruijn *et al*. in 2016^15^. This study aimed to compare quantitative and qualitative fFN testing for predicting delivery within 7 days after testing in women with threatened PTB. Inclusion criteria for the EUFIS study were largely in accordance with those of the QUiPP study^13^. Participants were eligible if they had symptoms of preterm labor (more than three contractions per 30 min, minor vaginal blood loss, or abdominal or back pain) and were between 24 and 34 weeks' gestation with intact membranes (Table S1). Unlike the QUiPP cohort, women between 23 and 24 weeks' gestation were not eligible. The study was conducted between January 2013 and May 2014 in 10 tertiary perinatal centers in Europe (Austria, Belgium, Germany, The Netherlands and Switzerland), and included women who presented themselves directly at a participating tertiary center or who were referred to the tertiary center by a secondary gynecologist, primary care midwife or general practitioner.

The results of qfFN testing (using the Rapid fFN 10Q analyzer; Hologic, Marlborough, MA, USA) and the shortest CL (measured by transvaginal ultrasound) were recorded. In contrast to the selection criteria for the QUiPP study^13^, sexual intercourse within 24 h before testing was not considered as an exclusion criterion, as Bruijn *et al*.^15^ found no association with qfFN value. Women were not included if tocolysis was initiated more than 18 h before testing. Moreover, patients were excluded when there was a contraindication for tocolysis, such as suspected intrauterine infection, acute maternal distress (severe vaginal blood loss or placental abruption), lethal congenital abnormality or suspected fetal distress.

Information on the presence of risk factors (twin pregnancy, history of sPTB, history of PPROM) was retrieved from the patients' medical records. No information was available on the history of cervical surgery. In order to use the QUiPP App v.2, we performed two analyses. In the first analysis, we assigned a value of zero to all patients for this variable, indicating that none had undergone cervical surgery. In the second analysis, we assigned a value of one to all patients, indicating that all had undergone cervical surgery. We expected an underestimation of the risk of sPTB in the former analysis, and the opposite in the latter.

**Figure 1:**
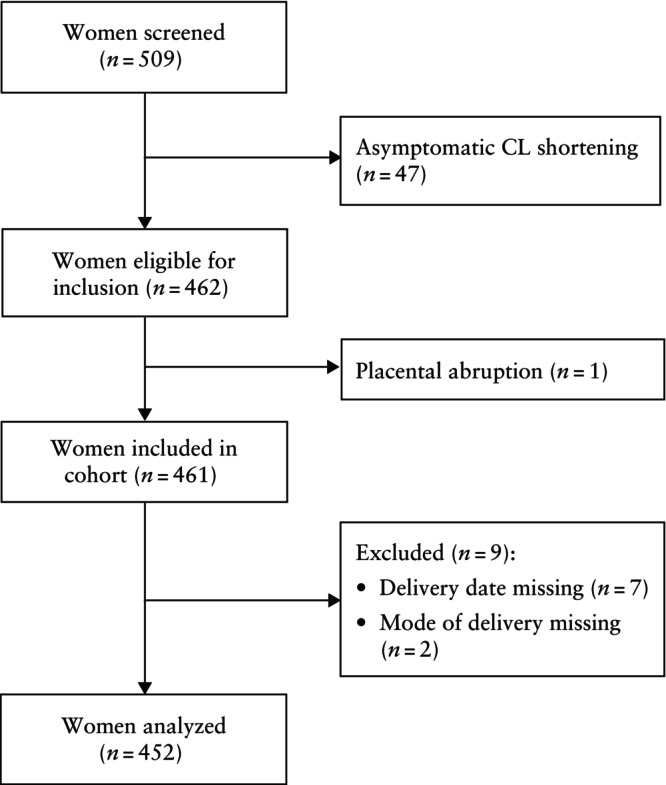
Flowchart summarizing inclusion of women in European Fibronectin Study cohort^15^. CL, cervical length.

The EUFIS cohort consisted of 509 women, of whom 452 were analyzed (Figure 1). The baseline characteristics of the EUFIS dataset are shown in Table 1. In this cohort, 449 CL measurements and 450 qfFN tests were available. In terms of risk factors, 9.1% of participants had a history of sPTB and 14.8% had a twin pregnancy. Data on previous PPROM were missing for 230 participants, and the choice was made to assign these participants a value of zero; as a result, we expect a slight underestimation of the risk of sPTB for these cases.

**Table 1:** Risk factors for spontaneous preterm birth (sPTB) used in QUiPP App v.2 and baseline characteristics of internal validation dataset (QUiPP cohort^13^) and three external validation datasets

Parameter	QUiPP (*n* = 506)	EUFIS (*n* = 452)	AUMC (*n* = 581)	UZG (*n* = 399)
QUiPP App v.2 risk factors				
History of cervical surgery	26 (5.1)	—	40 (6.9)	31 (7.8)
History of sPTB < 37 weeks	83 (16.4)	41 (9.1)	122 (21.0)	59 (14.8)
History of PPROM < 37 weeks	34 (6.7)	4/222 (1.8)	51/571 (8.9)	—
Twin pregnancy	33 (6.5)	67 (14.8)	102 (17.6)	84 (21.1)
GA at CL measurement and/or qfFN test (weeks)	—	29.6 (26.8–31.6)	28.4 (26.1–30.4)	28.57 (25.9–30.7)
CL (mm)	—	21.2 (15.0–27.0)†	16.0 (9.0–23.0)§	15.0 (9.0–21.0)
qfFN (ng/mL)	—	128.4 (8.0–215.8)‡	167.0 (66.5–331.2)¶	—
Other characteristics				
Maternal age (years)	29.8 ± 6.0	29.5 ± 5.2	30.3 ± 5.4	30.2 ± 4.9
Prepregnancy BMI (kg/m^2^)	26.0 ± 6.1*	23.5 ± 4.7	23.9 ± 4.6	23.6 ± 4.7
Cerclage	—	6 (1.3)	35 (6.0)	24 (6.0)
Pessary	—	37 (8.2)	24 (4.1)	10 (2.5)
Progesterone	—	91 (20.1)	127 (21.9)	242 (60.7)**
GA at birth (weeks)	—	37.9 (34.9–39.4)	34.7 (29.9–38.1)	32.3 (29.6–36.9)††
Nulliparous	—	247 (54.6)	340 (58.5)	244 (61.2)

Data are given as *n* (%), mean ± SD, *n*/*N* (%) or median (interquartile range).

* Unknown whether before pregnancy or at admission.

† Data available for 449 patients.

‡ Data available for 450 patients.

§ Data available for 580 patients.

¶ Data available for 212 patients.

** Progesterone was given at 12–16 weeks, 19–26 weeks or after starting tocolysis.

†† Data available for 288 patients. AUMC, Amsterdam University Medical Center; BMI, body mass index; CL, cervical length; GA, gestational age; EUFIS, European Fibronectin Study^15^; PPROM, preterm prelabor rupture of membranes; qfFN, quantitative fetal fibronectin; UZG, Ghent University Hospital.

#### Amsterdam University Medical Center

The second dataset used for external validation of the QUiPP App v.2 was retrieved retrospectively from the electronic health records of patients admitted to the Amsterdam University Medical Center (AUMC), Amsterdam, The Netherlands. The qfFN test was introduced in 2016, and therefore, data collection spans from January 2016 to November 2023. Within this timeframe, all medical records identified with a billing code indicative of threatened PTB underwent screening for eligibility, guided by the inclusion criteria of the QUiPP study^13^. All risk factors required for the QUiPP App v.2, along with supplementary well‐documented maternal risk factors, were gathered systematically by two authors (A.M.F. and A.L.R.). Inclusion and exclusion criteria are detailed in Table S1.

**Figure 2:**
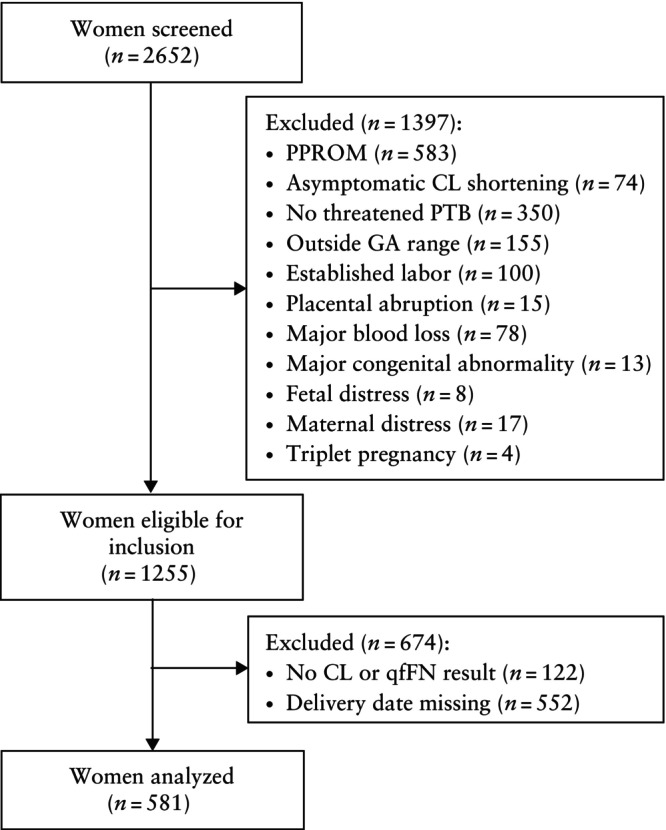
Flowchart summarizing inclusion of women in Amsterdam University Medical Center cohort. CL, cervical length; GA, gestational age; PPROM, preterm prelabor rupture of membranes; PTB, preterm birth; qfFN, quantitative fetal fibronectin.

A total of 2652 patients admitted to hospital for threatened PTB underwent screening for eligibility (Figure 2). In total, 1255 patients met the eligibility criteria. The main reason for ineligibility was PPROM (*n* = 583). If a patient experienced multiple hospital admissions for threatened PTB within the same pregnancy, only the initial admission was included in the dataset. If a patient had multiple admissions for threatened PTB across different pregnancies, each initial admission was considered. From the pool of eligible admissions, 122 were excluded because of missing CL or qfFN data. These were predominantly patients with bulging membranes, which precluded CL measurement. Missing qfFN data were attributable either to invalid test results or failure to document the quantitative result in the electronic health record. For 552 eligible women, the date of delivery was unknown; consequently, these patients were excluded from analysis and not subjected to statistical correction. As in the EUFIS dataset, sexual intercourse within 24 h before testing was not associated with qfFN level, so these patients were not excluded. In total, 581 admissions were analyzed.

The baseline characteristics of the AUMC dataset are outlined in Table 1. In this cohort, 580 CL measurements and 212 qfFN test results were available. Regarding risk factors, 21.0% of participants had a history of sPTB, 17.6% had a twin pregnancy, 6.9% had a history of cervical surgery and 8.9% had a history of PPROM before 37 weeks' gestation. For history of PPROM, data were missing in 10 cases, and similarly to the EUFIS dataset, the choice was made to assign these cases a value of zero.

#### Ghent University Hospital

**Figure 3:**
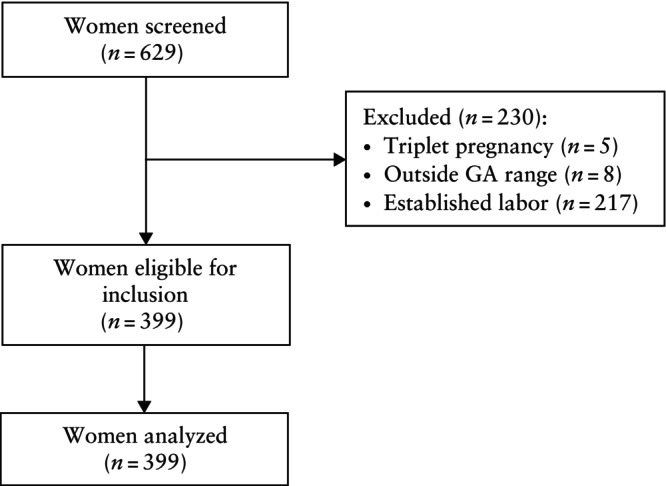
Flowchart summarizing inclusion of women in Ghent University Hospital cohort. GA, gestational age.

The third dataset used for the external validation of the QUiPP App v.2 was obtained from the preterm birth database of Ghent University Hospital (UZG), Ghent, Belgium. Patients hospitalized due to threatened PTB provided consent for their data to be included in a database dedicated to PTB research. The dataset includes records from 1 January 2012 to 30 April 2023. From 2012 until mid‐2017, the dataset comprised only women who delivered before 34 weeks' gestation. From mid‐2017 onwards, all patients with threatened sPTB were included. Of these patients, 66.9% were referred from secondary to tertiary care, of whom 44.9% were discharged home or back to the referring hospital, mostly at 32–34 weeks' gestation. As a result of the absence of a qfFN analyzer, only the QUiPP model using solely CL could be validated in this cohort. The exclusion criteria were triplets or higher‐order multiple pregnancy, gestational age at testing outside the permitted range, established labor, PPROM and severe fetal congenital malformation, defined as having an influence on neonatal morbidity and mortality (Table S1). A total of 399 patients met the eligibility criteria and were analyzed (Figure 3). For 288 of these patients, the date of birth was known, while for the remaining 111 patients, only the date of discharge was known, indicating that these patients were pregnant up to the discharge date. In survival analyses, these patients were classified as censored from the discharge date onwards. In this context, because censoring is related to the risk profile and/or gestational age of the patient, the reason for censoring is not random but informative and should be accounted for to correct for selection bias. We applied statistical corrections for patients with an unknown date of birth, as detailed below.

The baseline characteristics of the UZG dataset are presented in Table 1. Overall, 14.8% had a history of sPTB, 21.1% had a twin pregnancy and 7.8% had previous cervical surgery. Data on the history of PPROM < 37 weeks were missing for all patients; these cases were assigned a value of zero and the opposite scenario (value of one) was also analyzed.

### Ethics

The Medical Ethics Review Committee of AUMC gave approval for this study on 22 August 2023 (reference number 2023.0445) and waived the need for informed consent for the retrospective collection of patient data from the AUMC database. Written informed consent was obtained from all patients in the EUFIS study and UZG database. A data transfer agreement was established between UZG and AUMC.

### Clinical management of threatened preterm birth

The clinical management of threatened PTB differed slightly between the three cohorts and the original QUiPP cohort^13^. In the original QUiPP cohort, management was in accordance with the UK National Institute for Health and Care Excellence (NICE) preterm birth guideline^16^, which recommends a ‘treat‐all’ approach for women presenting with suspected preterm labor before 30 weeks' gestation. At or beyond 30 weeks, tocolysis and antenatal corticosteroids for fetal lung maturation were advised for women with CL < 15 mm or a positive fFN test (> 50 ng/mL).

In contrast, the EUFIS, AUMC and UZG cohorts did not distinguish between women before and after 30 weeks' gestation. In these cohorts, before 34 weeks' gestation, tocolysis and antenatal corticosteroids would be advised for women with CL < 15 mm, as well as for those with CL of 15–30 mm combined with a positive fFN test or positive phosphorylated insulin‐like growth factor‐binding protein (ph‐IGFBP) test (in the case of UZG). For women with CL of 15–30 mm and a negative fFN test, steroids and tocolysis were generally not administered in the AUMC and UZG cohorts and patients were not hospitalized, according to local protocol^17^, ^18^. In the EUFIS cohort, decisions regarding the initiation of tocolysis and steroids in these cases were left to the discretion of the on‐call clinician^15^.

### Statistical analysis

We evaluated risk prediction for sPTB using the three QUiPP App v.2 models: CL alone, qfFN alone and CL plus qfFN (formulae are provided in Appendix S1)^13^. The risk of sPTB was computed for each patient at enrollment and compared with the observed pregnancy outcome at six distinct time intervals: within 1, 2 and 4 weeks after testing, and at < 30, < 34 and < 37 weeks' gestation. Discriminative ability was evaluated by estimating the AUC. As a Delphi consensus survey recommended a range of 5–15% as the threshold for intervention for threatened PTB^19^, we calculated specificity, sensitivity and positive (LR+) and negative (LR−) likelihood ratios at fixed prediction rates (i.e. percentage of risk to indicate a positive test) of 5%, 10% and 15%. The clinical threshold in the original QUiPP study was set at 5%^13^. We performed 10 000 stratified bootstrap replicates to estimate 95% CI for all diagnostic test characteristics.

For the UZG database, we addressed the selection bias caused by loss to follow‐up of 111 patients (informative censoring) by applying inverse probability of censoring weighting (IPCW). This correction was necessary because the timing of censoring is related to the patient's risk profile and/or gestational age. Specifically, patients who are censored because of hospital discharge are less likely to have experienced the event of interest, i.e. PTB. As a result, the event is more frequently observed in patients who remain hospitalized (and are not censored), leading to biased survival probability estimates, because the observed event times are not representative of the entire population. IPCW compensates for this bias by assigning additional weight to non‐censored individuals who share similar characteristics with the censored patients^20^, ^21^, ^22^. These weights were estimated using a Cox proportional hazards model^23^, incorporating the same risk factors as those used in the QUiPP App v.2, as well as whether the patient was transferred from secondary care. This variable is highly predictive of PTB, as patients are often transferred only when the referring physician assesses that delivery is imminent. Patients at lower risk are typically managed with tocolysis in secondary care hospitals. Equivalent test characteristics (e.g. AUC, sensitivity) were calculated using the IPCW weights to estimate the time‐dependent ROC curve^24^.

The sensitivity and specificity of the QUiPP App v.2 in the UZG dataset for predicting sPTB within 1 and 2 weeks was calculated using a weighted average across all gestational ages at admission. As each patient is admitted at a different gestational age, the time horizons for the 1‐ and 2‐week predictions vary accordingly. To derive overall sensitivity and specificity values, we computed these metrics at each gestational age and applied a weighted average, where the weights corresponded to the proportion of patients admitted at each gestational age. Consequently, gestational timepoints associated with more admissions (e.g. 30 + 0 weeks) contributed more heavily to the final estimates compared with gestational ages associated with fewer admissions (e.g. 24 + 0 weeks). The LR+ and LR− were calculated using these final estimates of sensitivity and specificity.

Subsequently, the QUiPP prediction models were evaluated using calibration analysis to determine how well the predicted probabilities corresponded to the observed outcomes^25^. Accurate risk predictions are essential to prioritize intervention for high‐risk patients and avoid unnecessary treatment for low‐risk patients. Calibration plots were created by dividing the data into 10 bins, ranging from 0% to 100% with intervals of 10%. The mean predicted risk within each bin was calculated and plotted against the observed mean prevalence of all samples within that bin.

Model calibration was evaluated quantitatively by calculating the calibration‐in‐the‐large, calibration slope and calibration intercept. The calibration slope assesses the spread of estimated risks, with a target value of 1; a slope < 1 suggests overly extreme risk estimates, i.e. too high for high‐risk patients and too low for low‐risk patients, while a slope > 1 suggests the opposite. The calibration intercept has a target value of 0; negative values indicate overestimation of risk, while positive values indicate underestimation^25^.

The formulae of the QUiPP model were implemented in Python to obtain the risk estimates, and analyses were conducted using R statistical software (R Foundation for Statistical Computing, Vienna, Austria). The pROC^26^ package was used for discrimination and 95% CI evaluation, rms^27^ was used for calibration analysis, bootLR^28^ was used for LR calculation and RiskRegression^29^ was used for IPCW, time‐dependent AUC and 95% CI evaluation.

## RESULTS

**Figure 4:**
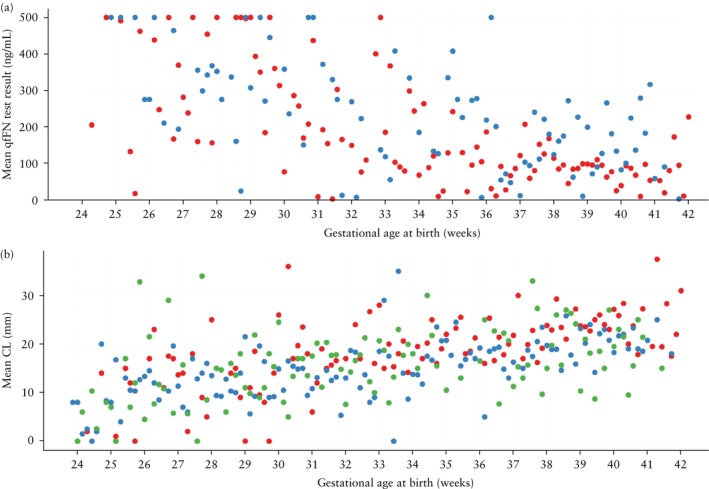
Mean quantitative fetal fibronectin (qfFN) test result (a) and mean cervical length (CL) (b) according to gestational age at birth, in European Fibronectin Study dataset^15^ (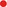), Amsterdam University Medical Center dataset (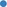) and Ghent University Hospital dataset (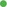). qfFN was not analyzed in the Ghent University Hospital dataset.

**Figure 5:**
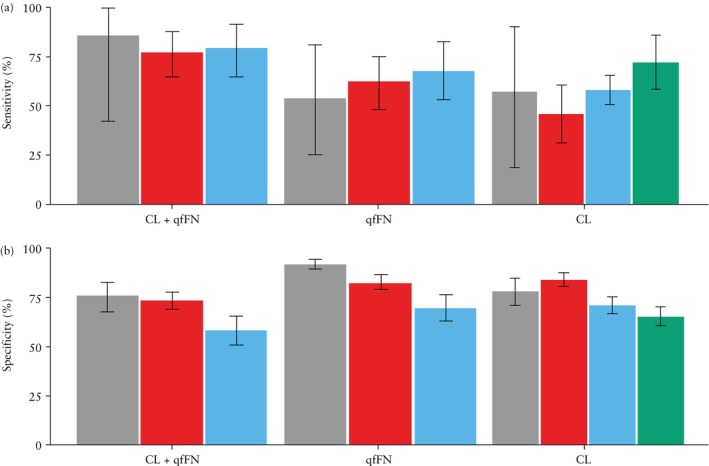
Sensitivity (a) and specificity (b) at 5% risk threshold, with 95% CI, for prediction by QUiPP App v.2 of risk of spontaneous preterm birth within 1 week after measurement of cervical length (CL) and/or quantitative fetal fibronectin (qfFN) in QUiPP internal validation dataset^13^ (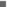), European Fibronectin Study dataset^15^ (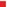), Amsterdam University Medical Center dataset (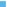) and Ghent University Hospital dataset (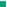).

A graphical representation of mean CL and mean qfFN level according to gestational age at delivery for all cohorts is shown in Figure 4. For all datasets, the results for the prediction of sPTB within 1 week after testing at risk thresholds of 5%, 10% and 15% are presented in the main text; all other results are included in the Supporting information. Sensitivity and specificity at a 5% risk threshold for all cohorts are shown in Figure 5. For comparison, the results obtained in the original QUiPP study^13^ are summarized in Appendix S1.

### European Fibronectin Study dataset

In the EUFIS cohort, the highest performance of the QUiPP App v.2 for the prediction of sPTB within 1 week after testing was achieved by the combination of CL and qfFN measurements (Table 2). The AUC was 0.84 (95% CI, 0.79–0.89). Sensitivity was 77.1% (95% CI, 64.6–87.5%) at a risk threshold of 5% and decreased to 43.8% (95% CI, 29.2–58.3%) at a risk threshold of 15%. The corresponding specificities were 73.4% (95% CI, 69.2–77.7%) and 92.0% (95% CI, 89.2–94.5%) (Table 3). LR+ and LR− were 2.90 (95% CI, 2.29–3.62) and 0.31 (95% CI, 0.16–0.49), respectively, at a risk threshold of 5%, and 5.46 (95% CI, 3.35–8.75) and 0.61 (95% CI, 0.46–0.76), respectively, at a risk threshold of 15%. The performance of the model based solely on qfFN was similar to that of the combined model. The ROC curves for all models and outcomes are provided in Figures S1–S3.

**Table 2:** Discrimination and calibration of QUiPP App v.2 for prediction of spontaneous preterm birth within 1 week after measurement of cervical length (CL) and/or quantitative fetal fibronectin (qfFN) in internal validation dataset (QUiPP cohort^13^) and two external validation datasets

				Sensitivity (%)			Calibration	
Model/dataset	Number of tests (*n*)	Events (*n* (%))	AUC	5% risk threshold	10% risk threshold	15% risk threshold	Intercept	Slope
CL + qfFN								
QUiPP	143	7 (4.9)	0.88 (0.77–0.98)	85.7 (42.1–99.6)	—	—	—	—
EUFIS	447	48 (10.7)	0.84 (0.79–0.89)	77.1 (64.6–87.5)	54.2 (41.6–66.7)	43.8 (29.2–58.3)	0.99	1.05
AUMC	212	34 (16.0)	0.74 (0.66–0.83)	79.4 (64.7–91.2)	55.9 (38.2–73.5)	29.4 (14.7–44.1)	0.63	0.83
qfFN only								
QUiPP	576	13 (2.3)	0.89 (0.81–0.98)	53.8 (25.1–80.8)	—	—	—	—
EUFIS	450	48 (10.7)	0.83 (0.78–0.88)	62.5 (47.9–77.1)	41.7 (29.2–56.3)	25.0 (12.5–37.5)	0.86	0.79
AUMC	212	34 (16.0)	0.72 (0.64–0.80)	67.6 (52.9–82.4)	35.3 (20.6–50.0)	17.6 (5.9–32.4)	0.28	0.58
CL only								
QUiPP	155	7 (4.5)	0.70 (0.45–0.95)	57.1 (18.4–90.1)	—	—	—	—
EUFIS	449	48 (10.7)	0.73 (0.65–0.80)	45.8 (31.3–60.4)	22.9 (12.5–35.4)	12.5 (4.2–22.9)	0.50	0.72
AUMC	580	162 (27.9)	0.71 (0.66–0.75)	58.0 (50.6–65.4)	30.2 (22.8–37.0)	13.6 (8.6–19.1)	1.31	0.63

Data in parentheses are 95% CI, unless stated otherwise. AUC, area under the receiver‐operating‐characteristics curve; AUMC, Amsterdam University Medical Center; EUFIS, European Fibronectin Study^15^.

**Table 3:** Specificity and positive (LR+) and negative (LR−) likelihood ratios for prediction by QUiPP App v.2 of spontaneous preterm birth within 1 week after measurement of cervical length and quantitative fetal fibronectin (qfFN)* in an internal validation dataset (QUiPP cohort^13^) and three external validation datasets

	Specificity (%)			LR+		LR−	
Dataset	5% risk threshold	10% risk threshold	15% risk threshold	5% risk threshold	15% risk threshold	5% risk threshold	15% risk threshold
QUiPP	75.7 (67.6–82.7)	—	—	3.53 (2.31–5.40)	—	0.19 (0.03–1.16)	—
EUFIS	73.4 (69.2–77.7)	84.5 (80.7–88.0)	92.0 (89.2–94.5)	2.90 (2.29–3.62)	5.46 (3.35–8.75)	0.31 (0.16–0.49)	0.61 (0.46–0.76)
AUMC	58.4 (51.1–65.7)	77.0 (70.9–83.2)	89.9 (85.4–93.8)	1.91 (1.47–2.43)	2.91 (1.31–5.67)	0.35 (0.14–0.62)	0.79 (0.60–0.95)
UZG†	65.4 (60.5–70.2)	75.3 (69.7–80.9)	81.6 (75.8–87.3)	2.08 (1.65–2.63)	2.54 (1.65–3.89)	0.43 (0.26–0.70)	0.65 (0.50–0.86)

Data in parentheses are 95% CI.

* qfFN was not measured at University Hospital Ghent (UZG).

† To estimate 95% CI for LRs, we applied log transformation and calculated the standard error of the log‐transformed LR, followed by exponentiation^35^. AUMC, Amsterdam University Medical Center; EUFIS, European Fibronectin Study^15^.

Overall, the QUiPP App demonstrated superior performance in predicting short‐term outcomes (i.e. sPTB within 1 week after testing, within 2 weeks after testing and at < 30 weeks' gestation) compared with long‐term outcomes (i.e. sPTB < 37 weeks' gestation) (Tables S2–S5). The highest AUC was 0.91 (95% CI, 0.86–0.95), which was obtained in the subgroup of pregnancies with sPTB at < 30 weeks' gestation, using the model based on both CL and qfFN (Table S3).

Setting the value of history of cervical surgery to one for all patients had a negligible effect on AUC values. However, the effect on sensitivity and specificity for the prediction of sPTB within 1 week after testing using the combined CL plus qfFN model was considerable, changing to 91.7% (95% CI, 83.3–97.9%) and 50.6% (95% CI, 45.6–55.6%), respectively, at a risk threshold of 5%.

### Amsterdam University Medical Center dataset

The AUC was 0.74 (95% CI, 0.66–0.83) for pregnancies with sPTB within 1 week after testing using the QUiPP App v.2 model that combined CL and qfFN measurements (Table 2). Consistent with the EUFIS dataset, the lowest AUC was observed in the model based solely on CL measurements. Sensitivity for sPTB within 1 week after testing using the combined CL plus qfFN model was 79.4% (95% CI, 64.7–91.2%) at a 5% risk threshold, dropping to 29.4% (95% CI, 14.7–44.1%) at a 15% risk threshold. The corresponding specificities were 58.4% (95% CI, 51.1–65.7%) and 89.9% (95% CI, 85.4–93.8%) (Table 3). LR+ and LR− were 1.91 (95% CI, 1.47–2.43) and 0.35 (95% CI, 0.14–0.62), respectively, at a risk threshold of 5%, and 2.91 (95% CI, 1.31–5.67) and 0.79 (95% CI, 0.60–0.95), respectively, at a risk threshold of 15%. Results for all models and outcomes are summarized in Tables S6–S9 and ROC curves are provided in Figures S4–S6.

### Ghent University Hospital dataset

The performance of the QUiPP App v.2 for the UZG dataset, using the CL‐only model, is summarized in Tables 3 and 4. For the pregnancies that experienced sPTB within 1 week after testing (CL measurement), the AUC was 0.80 (95% CI, 0.75–0.85). At a risk threshold of 5%, the sensitivity was 72.1% (95% CI, 58.3–85.9%), decreasing to 46.8% (95% CI, 32.5–61.2%) at a risk threshold of 15%. The corresponding specificities were 65.4% (95% CI, 60.5–70.2%) and 81.6% (95% CI, 75.8–87.3%). LR+ and LR− were 2.08 (95% CI, 1.65–2.63) and 0.43 (95% CI, 0.26–0.70), respectively, at a risk threshold of 5%, and 2.54 (95% CI, 1.65–3.89) and 0.65 (95% CI, 0.50–0.86), respectively, at a risk threshold of 15%. Additionally, the performance for predicting short‐term outcomes was superior to that for predicting long‐term outcomes. The plot of time‐dependent AUCs is provided in Figure S7.

**Table 4:** Discrimination and calibration of QUiPP App v.2 for prediction of spontaneous preterm birth (sPTB) at six predefined timepoints using cervical length in Ghent University Hospital dataset

					Sensitivity (%)		
Outcome	Screened (*n*)	Uncensored (*n*)	Censored (*n*)	AUC	5% risk threshold	10% risk threshold	15% risk threshold
sPTB within 1 week*	399	288	111	0.80 (0.75–0.85)	72.1 (58.3–85.9)	56.7 (42.3–71.1)	46.8 (32.5–61.2)
sPTB within 2 weeks*	399	288	111	0.77 (0.71–0.82)	76.2 (65.3–87.1)	62.3 (50.3–74.4)	52.4 (40.3–64.5)
sPTB within 4 weeks*	399	288	111	0.66 (0.59–0.73)	75.8 (68.1–83.5)	64.9 (56.0–73.7)	55.8 (46.3–65.2)
sPTB < 30 weeks	266	180	86	0.62 (0.54–0.70)	69.9 (59.3–80.6)	55.5 (44.2–66.8)	49.1 (37.7–60.4)
sPTB < 34 weeks	399	288	111	0.60 (0.51–0.69)	88.2 (81.8–94.5)	77.6 (70.0–85.3)	66.7 (58.8–74.7)
sPTB < 37 weeks	399	288	111	0.54 (0.42–0.66)	99.7 (99.1–100)	96.0 (92.2–99.8)	90.9 (85.9–95.9)

Data in parentheses are 95% CI.

* After cervical length measurement. AUC, area under the receiver‐operating‐characteristics curve.

Setting the value of history of PPROM < 37 weeks to one for all patients had a negligible effect on AUC values. However, the impact on sensitivity and specificity for the prediction of sPTB within 1 week after testing using the CL‐only model was substantial, changing to 83.9% (95% CI, 72.4–95.4%) and 57.8% (95% CI, 53.1–62.5%), respectively, at a risk threshold of 5%.

### Calibration

**Figure 6:**
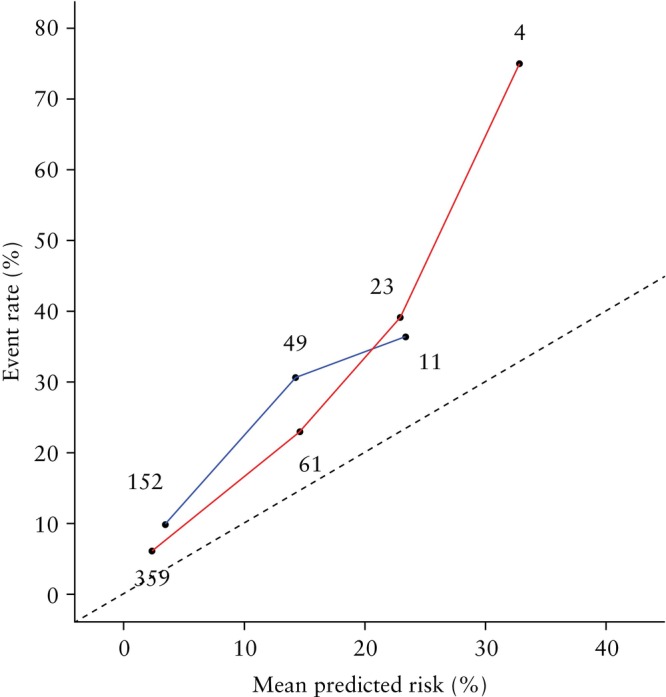
Calibration plot for prediction by QUiPP App v.2 of risk of spontaneous preterm birth within 1 week after measurement of cervical length and quantitative fetal fibronectin in European Fibronectin Study dataset^15^ (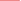) and Amsterdam University Medical Center dataset (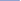). Numbers of samples are annotated. Dashed line indicates perfect calibration.

We analyzed the calibration plots for all outcomes and models (Figures S8–S13) but present here only the plots corresponding to the shortest time frame for which a model makes a prediction, i.e. sPTB within 1 week after testing. Given that this period typically aligns with critical clinical decisions, such as hospital admission or initiation of treatment, we argue that optimal calibration of risk predictions during this time frame is most relevant. Moreover, as the model incorporating both CL and qfFN had the best discriminative power for both the EUFIS and AUMC datasets (AUC of 0.84 and 0.74, respectively), we present the calibration plots for this model in Figure 6. Both plots lie above the line of perfect calibration, indicating that the event rate exceeds the mean predicted risk. In other words, the model tends to slightly underestimate the risk of sPTB within 1 week after testing. A significant majority of samples received risk predictions within the range of 0–10% (359 samples in the EUFIS dataset and 152 in the AUMC dataset). Quantitative calibration analysis determined an intercept of 0.63 (suggesting underestimation of risk) and a slope of 0.83 (indicating slightly exaggerated risk estimates) in the AUMC cohort (Table 2). In the EUFIS dataset, the trends were reversed, with an intercept of 0.99 and a slope of 1.05. The EUFIS dataset exhibited closer proximity to perfect calibration in terms of slope but poorer calibration in terms of intercept.

It was not possible to plot the corresponding calibration curve for the UZG dataset because the unknown status of censored patients meant that predicted risk could not be compared against the observed event rate.

## DISCUSSION

The best performing model for predicting sPTB within 1 week after testing using the QUiPP App v.2 was that combining CL and qfFN measurements, with an AUC of 0.84 (95% CI, 0.79–0.89) in the EUFIS dataset and 0.74 (95% CI, 0.66–0.83) in the AUMC dataset. Notably, the AUC for the EUFIS dataset is similar to that derived in the internal validation of the QUiPP App (0.88 (95% CI, 0.77–0.98))^13^. In the UZG dataset, the model using only CL measurement resulted in a time‐dependent AUC of 0.80 (95% CI, 0.75–0.85), using IPCW to correct for censored patients.

The calibration plots showed more nuance regarding the reliability of the models' risk predictions. As the level of predicted risk increased, there was a sharp decline in the number of samples receiving such predictions. This made it challenging to assess visually the calibration when comparing predicted risk with observed event rate. In the case of the EUFIS dataset, calibration appeared to be stronger around the risk threshold of 5%.

Similar to the external validation study of the QUiPP App v.2 conducted by Creswell *et al*.^30^, our research is constrained by the phenomenon known as the ‘treatment paradox’^31^. This paradox arises when a robust predictor for an outcome (e.g. a short cervix for predicting PTB^32^, ^33^, ^34^) leads to the implementation of an effective intervention, such as progesterone, which reduces the risk of PTB. Consequently, the strong predictor may appear less effective within a prediction model, as its impact has been mitigated by the intervention. This could explain why the QUiPP App model based only on CL performed worse across all three cohorts compared with the models that included qfFN, either alone or combined with CL.

### Strengths and limitations

This is the first study to validate externally the QUiPP App v.2 in a symptomatic population. The validation comprised three independent datasets, which included a large number of symptomatic patients referred to tertiary care over the period 2012–2023, and so contributes to the evidence base on the effectiveness of the QUiPP App v.2. Moreover, we studied the predictive value of the QUiPP App v.2 for cases with complete data (EUFIS and AUMC) and a dataset that was statistically corrected for censoring (UZG). The external validation of the QUiPP App v.2 by Creswell *et al*.^30^ was conducted in an asymptomatic high‐risk population. The study showed similar findings to ours with respect to accurate calibration of the risk of sPTB in low‐risk patients. However, it showed a trend towards overestimation of risk for women at higher risk of sPTB, whereas our results show, in general, an underestimation of risk for patients with higher risk estimates.

A significant limitation of our investigation is the presence of selection bias in the AUMC dataset. We had to omit 552 samples due to missing delivery date. The majority of these patients were discharged home and are presumed to have not delivered < 32 weeks' gestation or within 1 week after admission, as their likelihood of delivery at the AUMC hospital would otherwise have been high. If these 552 samples had been included, nearly doubling the size of this dataset, the characteristics of this population could have been altered substantially. Furthermore, we excluded an additional 122 samples because of missing CL measurement or qfFN test result, and these patients are likely to have a higher‐risk profile. We addressed selection bias caused by loss to follow‐up in the UZG cohort by applying IPCW to the entire dataset, encompassing both censored and uncensored patients. We observed that the time‐dependent AUC decreased when moving from short‐ to long‐term predictions, a trend that was also evident in the complete datasets (EUFIS and AUMC). A possible explanation for the better short‐term predictive performance is that the excluded patients were likely to have had longer pregnancy duration. All three datasets exhibit a form of selection bias in their populations. Ideally, the QUiPP App v.2 should be used to screen all symptomatic women who might be admitted for threatened PTB. However, in the AUMC and UZG datasets, only patients who required admission were included in the cohort.

Another limitation of this investigation pertains to the absence of data regarding the history of cervical surgery in the EUFIS dataset and history of PPROM in the UZG dataset. Given the unavailability of these variables, all women were assumed to be devoid of such history. In the formulation of all QUiPP models, the presence of one or more risk factors is assigned an equivalent risk score, thereby ignoring the cumulative effect of risk factors. Of 346 (77%) samples in the EUFIS dataset for which no risk factor was identified, the missing cervical history could potentially exert an influence. Carter *et al*.^13^ reported an overall prevalence of 5.9% for history of cervical surgery, indicating that the absence of this information could have led to underestimation of risk in 21 cases in the EUFIS dataset. Likewise, for previous PPROM, this could lead to underestimation of risk in 22 cases in the UZG dataset (7.0% prevalence in the study of Carter *et al*.^13^ and 314 patients without a risk factor).

### Conclusions

In this external validation study, the QUiPP App v.2 had high discriminative ability in the EUFIS dataset and moderate discriminative capability in the AUMC dataset using qfFN with or without CL in the short‐term prediction of sPTB. For patients with low predicted risk of sPTB in particular, the QUiPP App v.2 can offer reassurance to both patients and clinicians. The models exhibit better predictive performance for short‐term outcomes compared with late preterm births. The model using CL alone exhibited inferior accuracy in the EUFIS and AUMC datasets. The results from the UZG dataset containing both censored and uncensored patients demonstrated the importance of statistically correcting for patients lost to follow‐up. The discriminative performance of CL alone for the short‐term prediction of sPTB was moderate in cases with complete data (EUFIS and AUMC datasets) but good in the UZG dataset, where statistical correction was performed to account for loss to follow‐up.

However, clinicians should consider several factors when using the QUiPP App v.2. The QUiPP App v.2 comprises three distinct prediction models with varying AUC scores, yet the application does not display the AUC of each model. Although very low‐risk patients are identified accurately by the app, calibration for higher‐risk patients displays more unpredictable behavior and involves substantially fewer samples. Clinical judgment should remain the primary basis for decision‐making in high‐risk cases.

### Disclosures

A.M.F., A.L.R. and P.C.A.M.B. are funded by a grant of public–private partnerships (PPP) from Amsterdam University Medical Center (identifier 2007651). The collaboration project is cofunded by the PPP Allowance made available by Health~Holland, Top Sector Life Sciences & Health. This study was performed in the context of the COCOON (combining cord‐free uterine electrohysterography and standard clinical measurements for refining the detection of premature birth) Study, a cooperation of Stichting VUmc, Stichting VU, Health~Holland and Bloom Technologies NV. These funding bodies played no role in the preparation of this paper. B.W.M. reports consultancy, travel support and research funding from Merck and consultancy for Organon and Norgine. B.W.M. holds stock from ObsEva and is supported by a NHMRC Practitioner Fellowship (GNT1082548).

## Supporting information


**Appendix S1** Formulae for QUiPP App v.2 algorithms for symptomatic women (reproduced from Carter *et al*.^13^)

**Figures S1–S3** Receiver‐operating‐characteristics curves showing prediction by QUiPP App v.2 of risk of spontaneous preterm birth at six predefined timepoints using cervical length (CL) plus quantitative fetal fibronectin (qfFN) model (Figure S1), qfFN‐only model (Figure S2) and CL‐only model (Figure S3) in European Fibronectin Study dataset.
**Figures S4–S6** Receiver‐operating‐characteristics curves showing prediction by QUiPP App v.2 of risk of spontaneous preterm birth at six predefined timepoints using cervical length (CL) plus quantitative fetal fibronectin (qfFN) model (Figure S4), qfFN‐only model (Figure S5) and CL‐only model (Figure S6) in Amsterdam University Medical Center dataset.
**Figure S7** Time‐dependent area under the receiver‐operating‐characteristics curve for prediction by QUiPP App v.2 of risk of spontaneous preterm birth using only cervical length in Ghent University Hospital dataset.
**Figures S8–S10** Calibration plots for prediction by QUiPP App v.2 of risk of spontaneous preterm birth at six predefined timepoints using cervical length (CL) plus quantitative fetal fibronectin (qfFN) model (Figure S8), qfFN‐only model (Figure S9) and CL‐only model (Figure S10) in European Fibronectin Study dataset.
**Figures S11–S13** Calibration plots for prediction by QUiPP App v.2 of risk of spontaneous preterm birth at six predefined timepoints using cervical length (CL) plus quantitative fetal fibronectin (qfFN) model (Figure S11), qfFN‐only model (Figure S12) and CL‐only model (Figure S13) in Amsterdam University Medical Center dataset.

**Table S1** Inclusion and exclusion criteria for European Fibronectin Study (EUFIS) cohort, Amsterdam University Medical Center (AUMC) cohort, Ghent University Hospital (UZG) cohort and original QUiPP study
**Table S2** Positive and negative likelihood ratios for prediction by QUiPP App v.2 of risk of spontaneous preterm birth at six predefined timepoints using cervical length plus quantitative fetal fibronectin model in European Fibronectin Study dataset
**Tables S3–S5** Discrimination and calibration of QUiPP App v.2 for prediction of spontaneous preterm birth at six predefined timepoints using cervical length (CL) plus quantitative fetal fibronectin (qfFN) model (Table S3), qfFN‐only model (Table S4) and CL‐only model (Table S5) in European Fibronectin Study dataset
**Table S6** Positive and negative likelihood ratios for prediction by QUiPP App v.2 of risk of spontaneous preterm birth at six predefined timepoints using cervical length plus quantitative fetal fibronectin model in Amsterdam University Medical Center dataset
**Tables S7–S9** Discrimination and calibration of QUiPP App v.2 for prediction of spontaneous preterm birth at six predefined timepoints using cervical length (CL) plus quantitative fetal fibronectin (qfFN) model (Table S7), qfFN‐only model (Table S8) and CL‐only model (Table S9) in Amsterdam University Medical Center dataset

## Data Availability

The data that support the findings of this study are available on request from the corresponding author. The data are not publicly available due to privacy or ethical restrictions.
