# Supplementary material for: External validation of QUiPP App in three independent European cohorts of symptomatic women
Source: Ultrasound Obstet Gynecol. 2025 Jun 26;66(2):163–74. doi: 10.1002/uog.29263 (PMC12317302; doi:10.1002/uog.29263)
Supplement: Supplementary file 1 — Appendix S1 Formulae for QUiPP App v.2 algorithms for symptomatic women (reproduced from Carter et al. 13 ) [file UOG-66-163-s002.docx]

# Appendix S1: Formulae for QUIPP App v.2 algorithms for symptomatic women (reproduced from Carter et al. ^13^)

### Symptomatic women with both CL and fFN measured:

Information used:

- Symptoms suggestive of abnormal or premature uterine activity
- Previous cervical surgery
- Previous PPROM
- Previous spontaneous preterm birth ≤36+6
- Number of fetuses
- Gestation of test (days)
- Shortest cervical length (mm)
- fFN result (ng/ml)

Mu = 0.00896*CL-0.0007748*fFN-0.1351627 (if twin pregnancy, previous cervical surgery, PPROM or preterm birth) + 5.53231915.

Sigma =.1744687

S(test) = 1-( Φ(log_e_(gestation of test-Mu)/Sigma)

(Where Φ is the Cumulative probability function of the Normal distribution)

S(30) = 1-( Φ (log_e_(30*7-Mu)/Sigma); S(34) S(37) are calculated similarly

S(1week) = 1-( Φ ( log_e_(gestation of test+1*7-Mu)/Sigma); likewise S(2 weeks) S(4 weeks)

Probability of delivering before 30 weeks = (S(test)-S(30))/S(test)

Other probabilities are calculated similarly

### Symptomatic women with only CL measured:

Mu = 2.397495*log_e_(CL+1)/10 -.1751846 (if twin pregnancy, previous cervical surgery, PPROM or preterm birth) + 4.9803773

Sigma = .2069215

All other results are calculated as method described in ‘symptomatic women with both CL and fFN measured’.

### Symptomatic women with only fFN measured:

Mu = -0.0013155*fFN -0.1911503 (if twin pregnancy, previous cervical surgery, PPROM or preterm birth) + 5.936622

Sigma = .22101474

All other results are calculated as method described in ‘symptomatic women with both CL and fFN measured’.
