# Supplementary material for: External validation of QUiPP App in three independent European cohorts of symptomatic women
Source: Ultrasound Obstet Gynecol. 2025 Jun 26;66(2):163–74. doi: 10.1002/uog.29263 (PMC12317302; doi:10.1002/uog.29263)
Supplement: Supplementary file 2 — Figures S1–S3 Receiver‐operating‐characteristics curves showing prediction by QUiPP App v.2 of risk of spontaneous preterm birth at six predefined timepoints using cervical length (CL) plus quantitative fetal fibronectin (qfFN) model (Figure S1), qfFN‐only model (Figure S2) and CL‐only model (Figure S3) in European Fibronectin Study dataset. Figures S4–S6 Receiver‐operating‐characteristics curves showing prediction by QUiPP App v.2 of risk of spontaneous preterm birth at six predefined timepoints using cervical length (CL) plus quantitative fetal fibronectin (qfFN) model (Figure S4), qfFN‐only model (Figure S5) and CL‐only model (Figure S6) in Amsterdam University Medical Center dataset. Figure S7 Time‐dependent area under the receiver‐operating‐characteristics curve for prediction by QUiPP App v.2 of risk of spontaneous preterm birth using only cervical length in Ghent University Hospital dataset. Figures S8–S10 Calibration plots for prediction by QUiPP App v.2 of risk of spontaneous preterm birth at six predefined timepoints using cervical length (CL) plus quantitative fetal fibronectin (qfFN) model (Figure S8), qfFN‐only model (Figure S9) and CL‐only model (Figure S10) in European Fibronectin Study dataset. Figures S11–S13 Calibration plots for prediction by QUiPP App v.2 of risk of spontaneous preterm birth at six predefined timepoints using cervical length (CL) plus quantitative fetal fibronectin (qfFN) model (Figure S11), qfFN‐only model (Figure S12) and CL‐only model (Figure S13) in Amsterdam University Medical Center dataset. [file UOG-66-163-s001.docx]

# Supplementary figures

This supplemental file contains the ROC curves for all models and subgroups:

- EUFIS dataset (Figures S1-S3)
- AUMC dataset (Figures S4-S6)
- UZ Gent dataset (time-dependent ROC curve, Figure S7)

The time-dependent ROC curve of the UZ Gent dataset shows the AUC scores along the gestational age. For example, if a patient is admitted at 29 + 0 gestation, one can check the AUC score at 30 + 0 gestation to see how good the model performs for the <1 week prediction. For the <2 weeks prediction one should check the score at 32 + 0.

The calibration plots for all models and subgroups:

- EUFIS dataset (Figures S8-S10)
- AUMC dataset (Figures S11-S13)


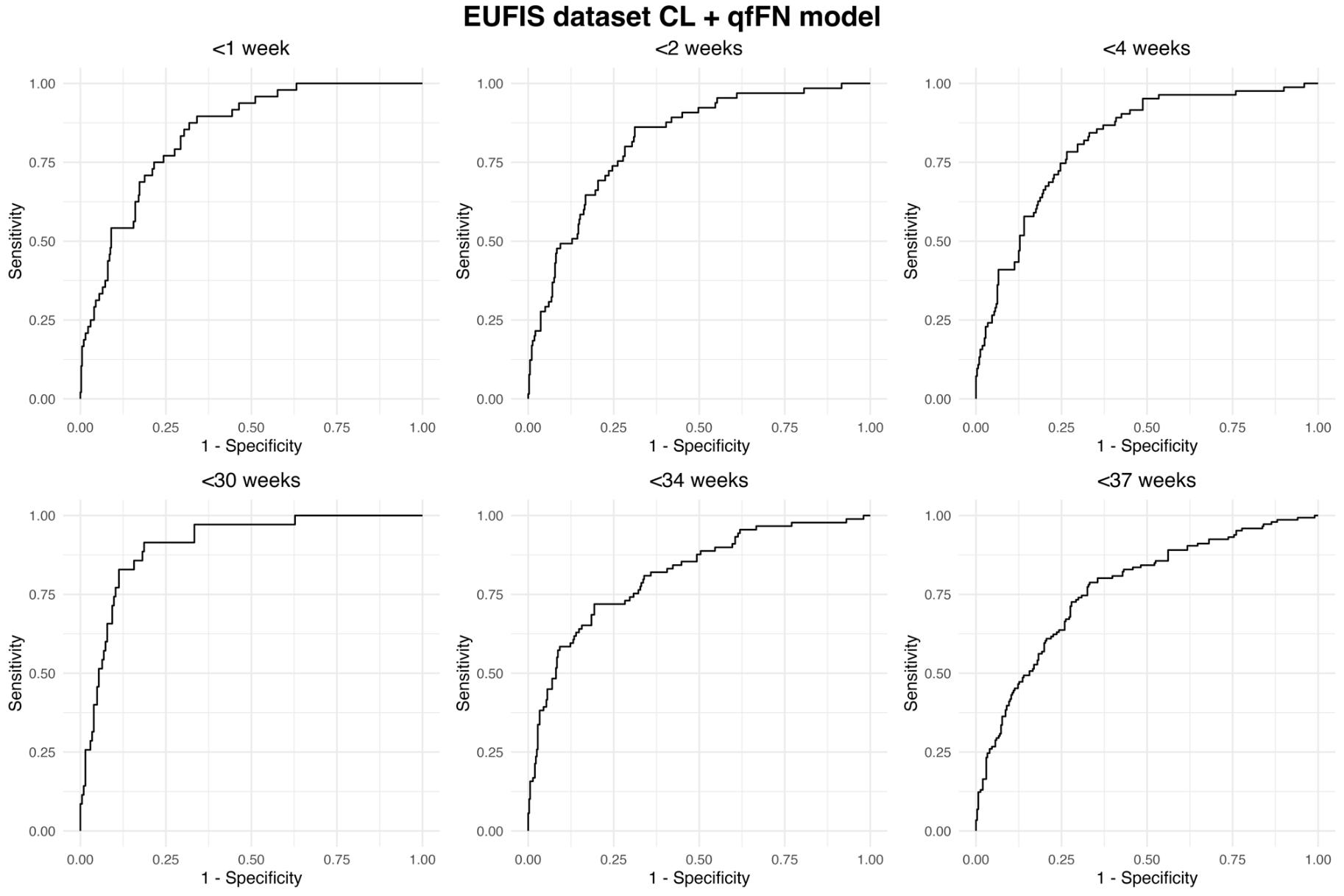


Figure S1: Receiver-operating-characteristics curves showing prediction by QUiPP App v.2 of risk of spontaneous preterm birth at six predefined timepoints using cervical length plus quantitative fetal fibronectin model in European Fibronectin Study dataset.


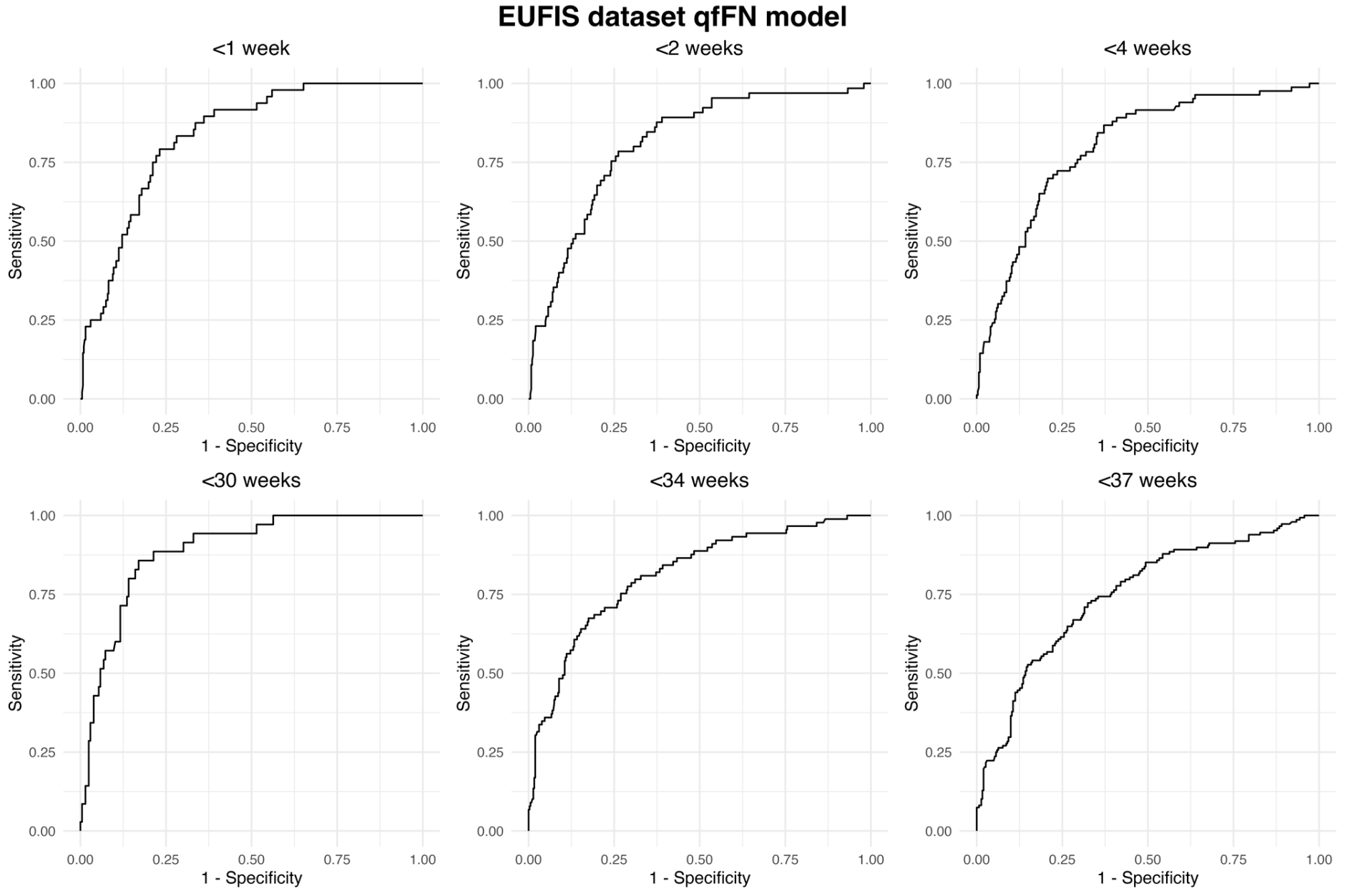


Figure S2: Receiver-operating-characteristics curves showing prediction by QUiPP App v.2 of risk of spontaneous preterm birth at six predefined timepoints using quantitative fetal fibronectin model in European Fibronectin Study dataset.


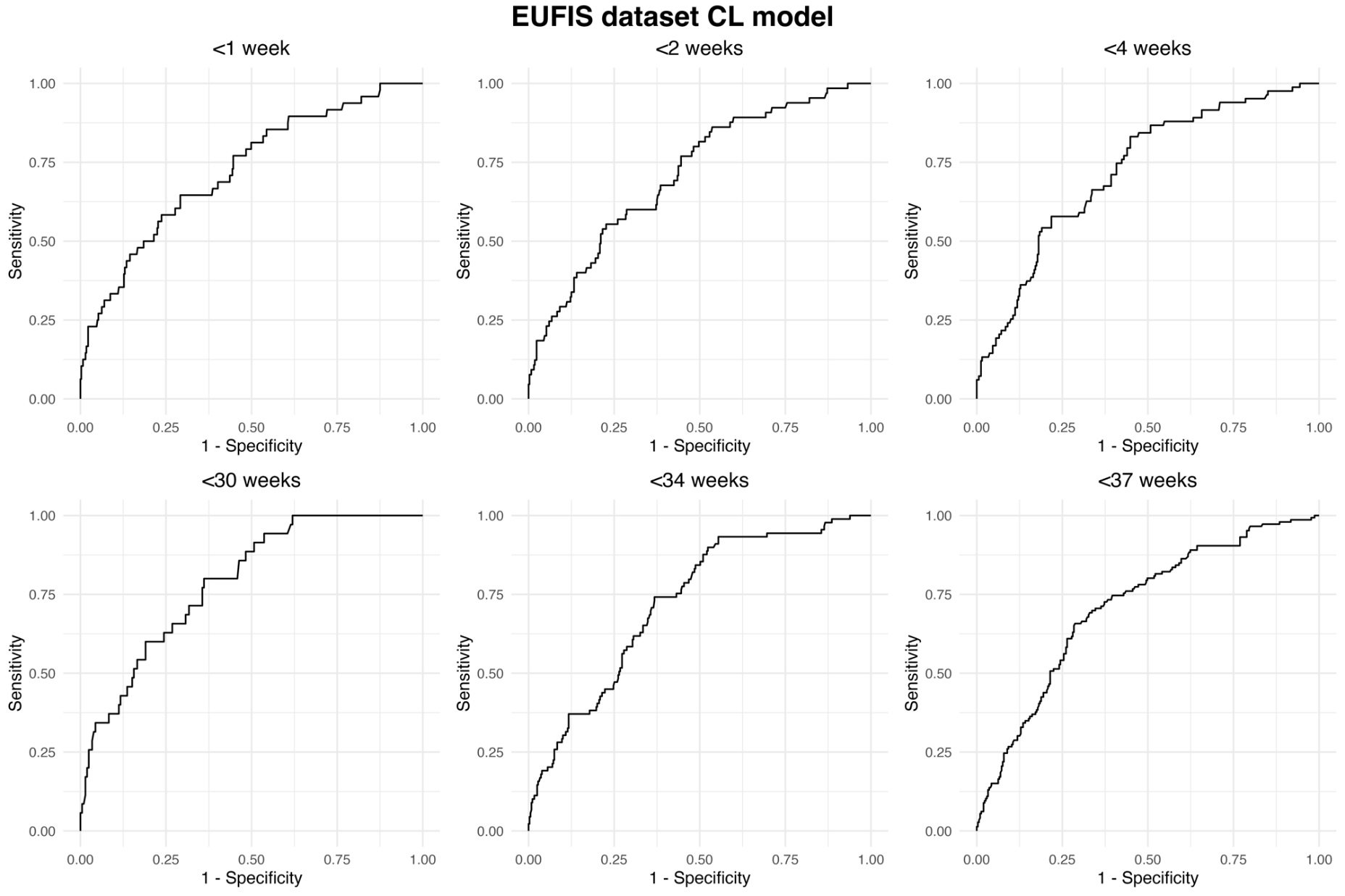


Figure S3: Receiver-operating-characteristics curves showing prediction by QUiPP App v.2 of risk of spontaneous preterm birth at six predefined timepoints using cervical length model in European Fibronectin Study dataset.


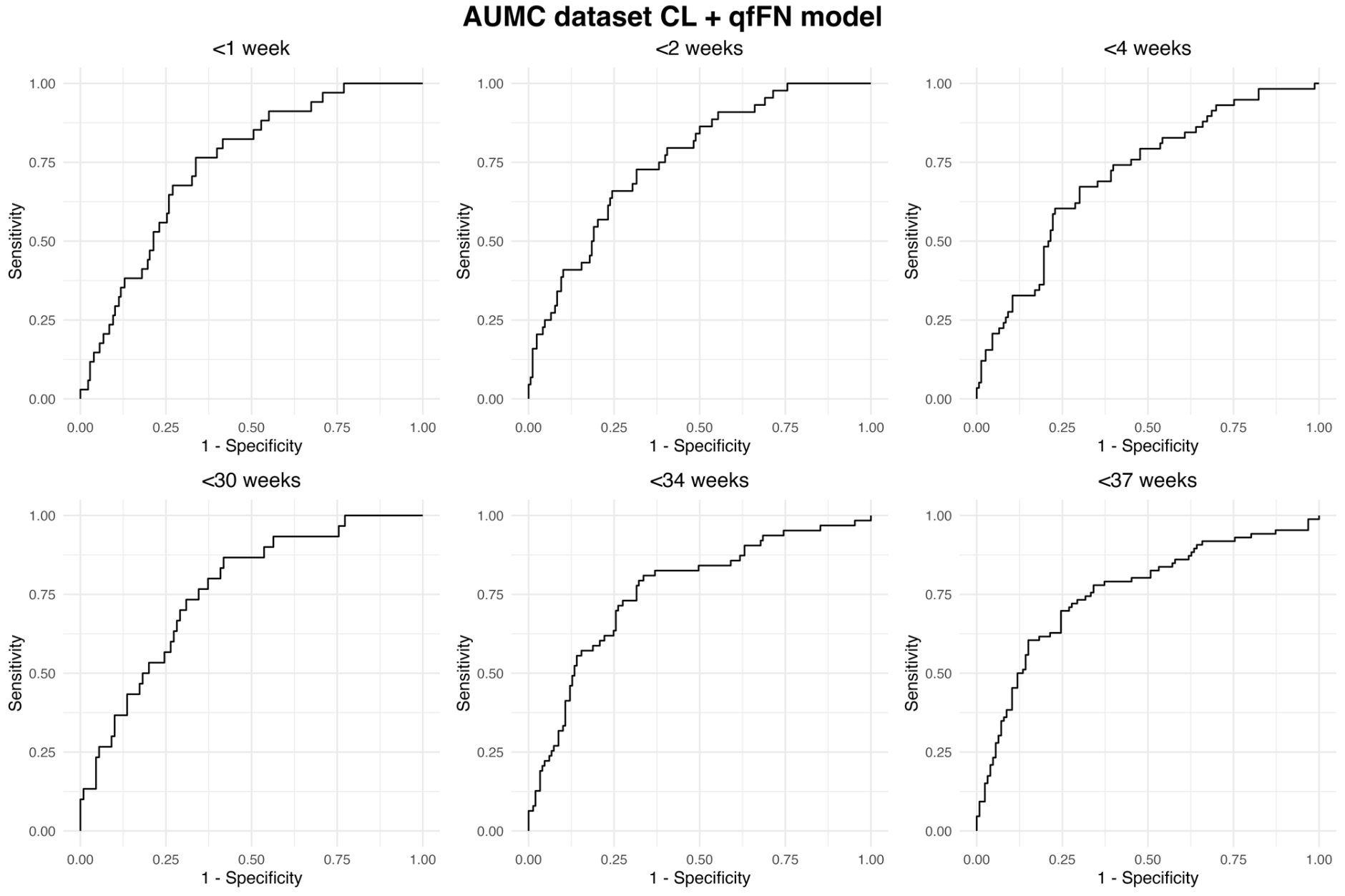


Figure S4: Receiver-operating-characteristics curves showing prediction by QUiPP App v.2 of risk of spontaneous preterm birth at six predefined timepoints using cervical length plus quantitative fetal fibronectin model in Amsterdam University Medical Centre dataset.


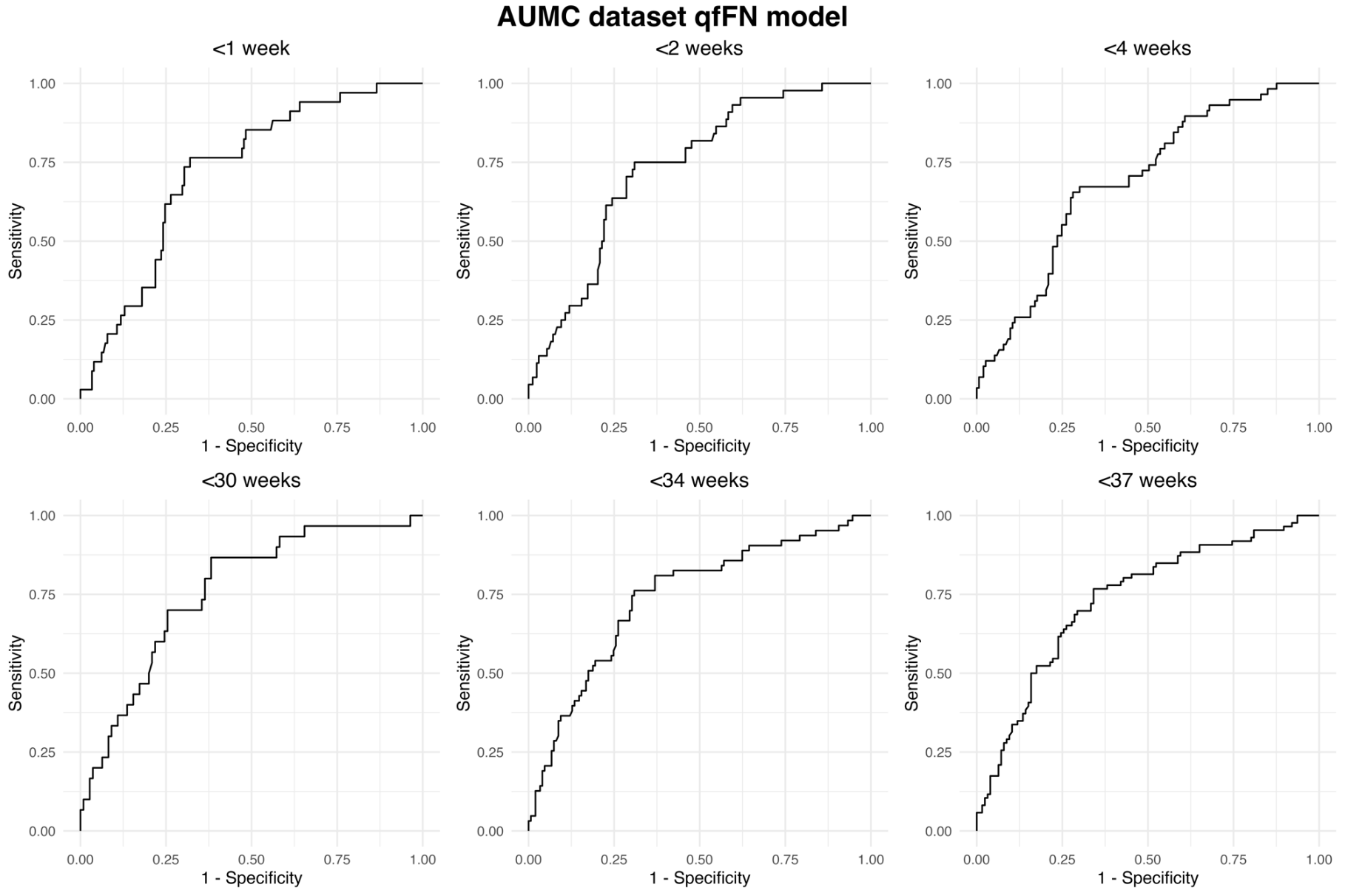


Figure S5: Receiver-operating-characteristics curves showing prediction by QUiPP App v.2 of risk of spontaneous preterm birth at six predefined timepoints using quantitative fetal fibronectin in Amsterdam University Medical Centre dataset.


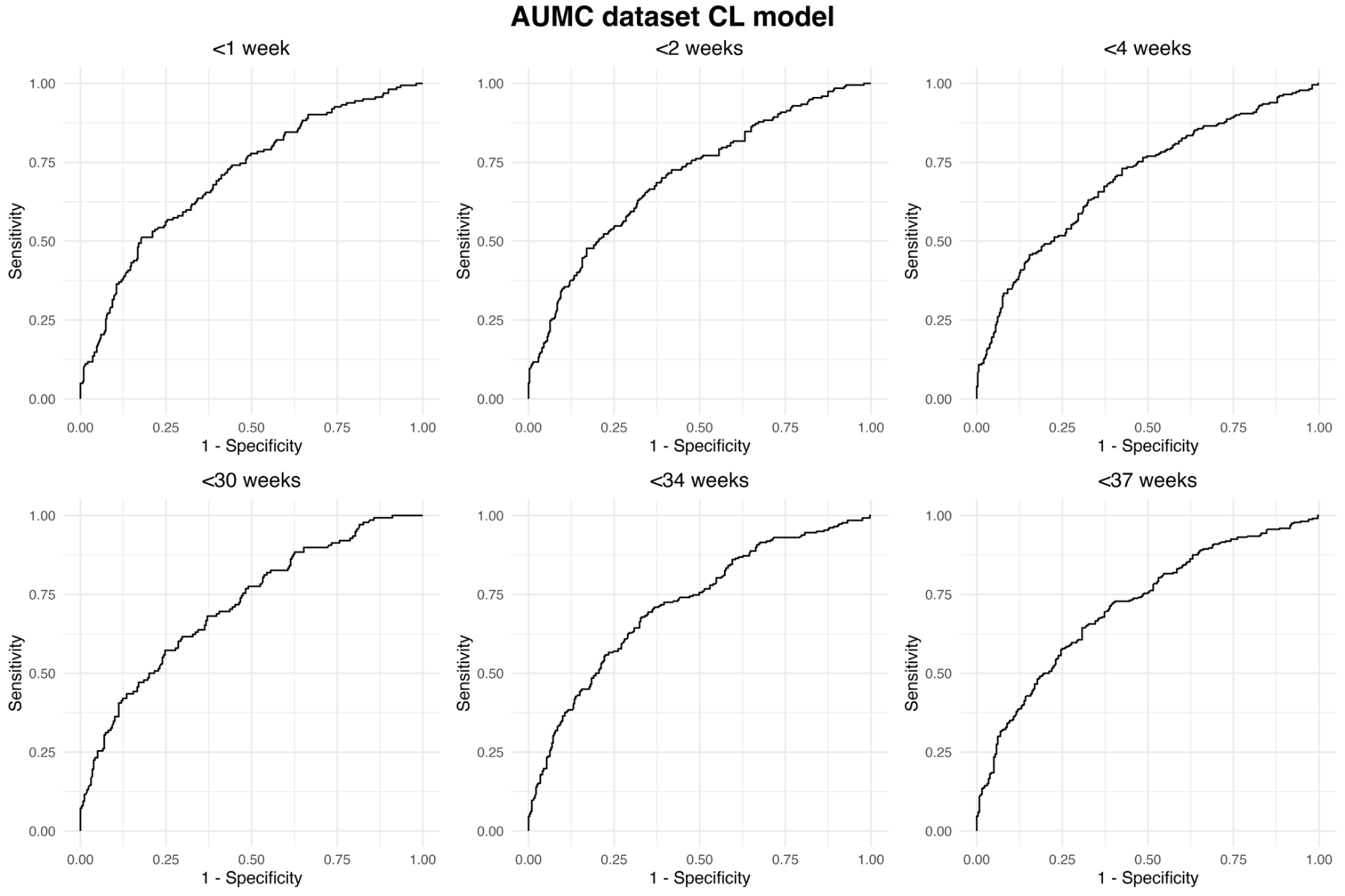


Figure S6: Receiver-operating-characteristics curves showing prediction by QUiPP App v.2 of risk of spontaneous preterm birth at six predefined timepoints using cervical length in Amsterdam University Medical Centre dataset.


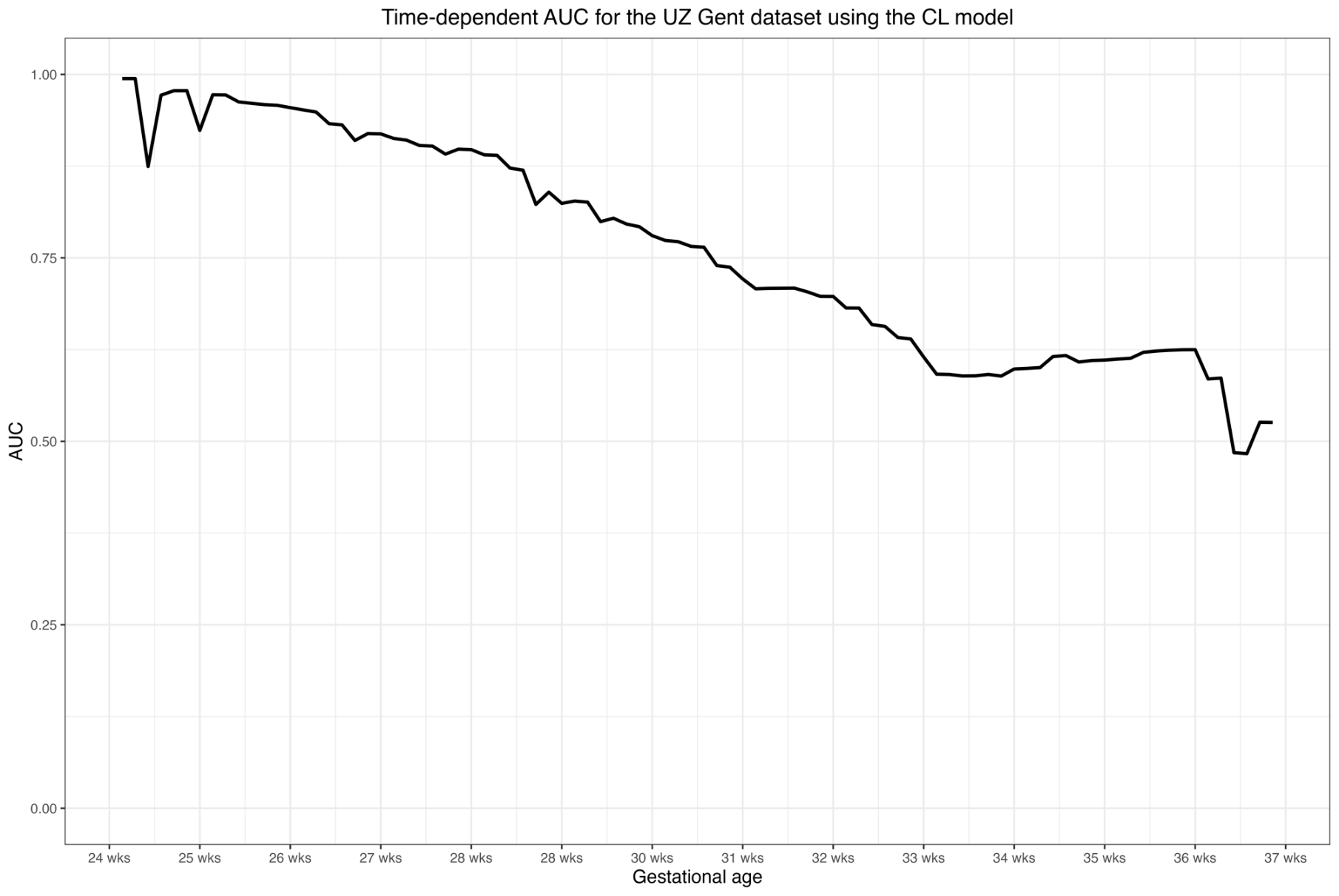


Figure S7: Time-dependent area under receiver-operating-characteristics curve for prediction by QuiPP App v.2 of risk of spontaneous preterm birth using only cervical length in Ghent University Hospital dataset.


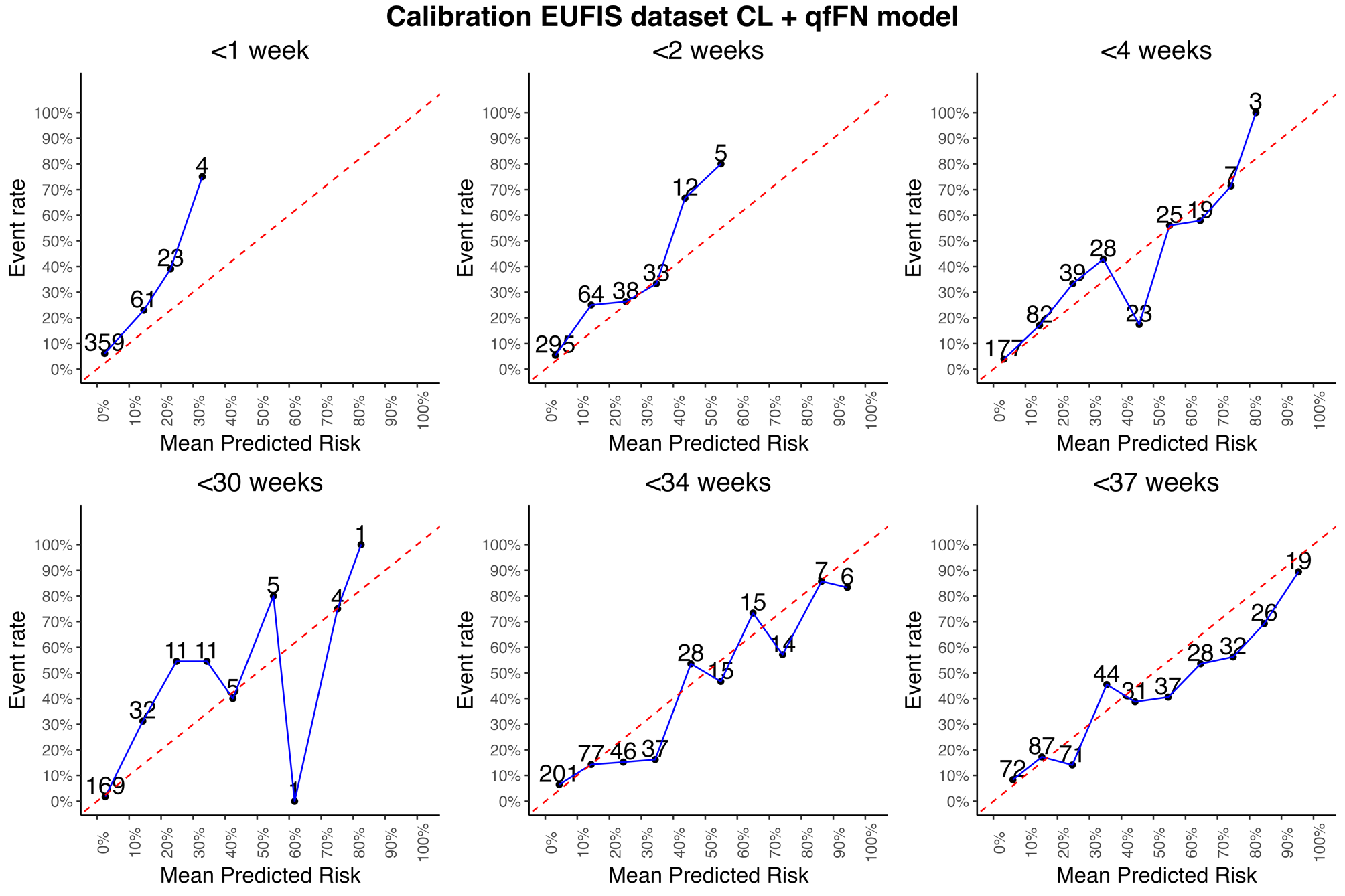


Figure S8: Calibration plots for prediction by QUiPP App v.2 of risk of spontaneous preterm birth at six predefined timepoints using cervical length plus quantitative fetal fibronectin in European Fibronectin Study dataset.


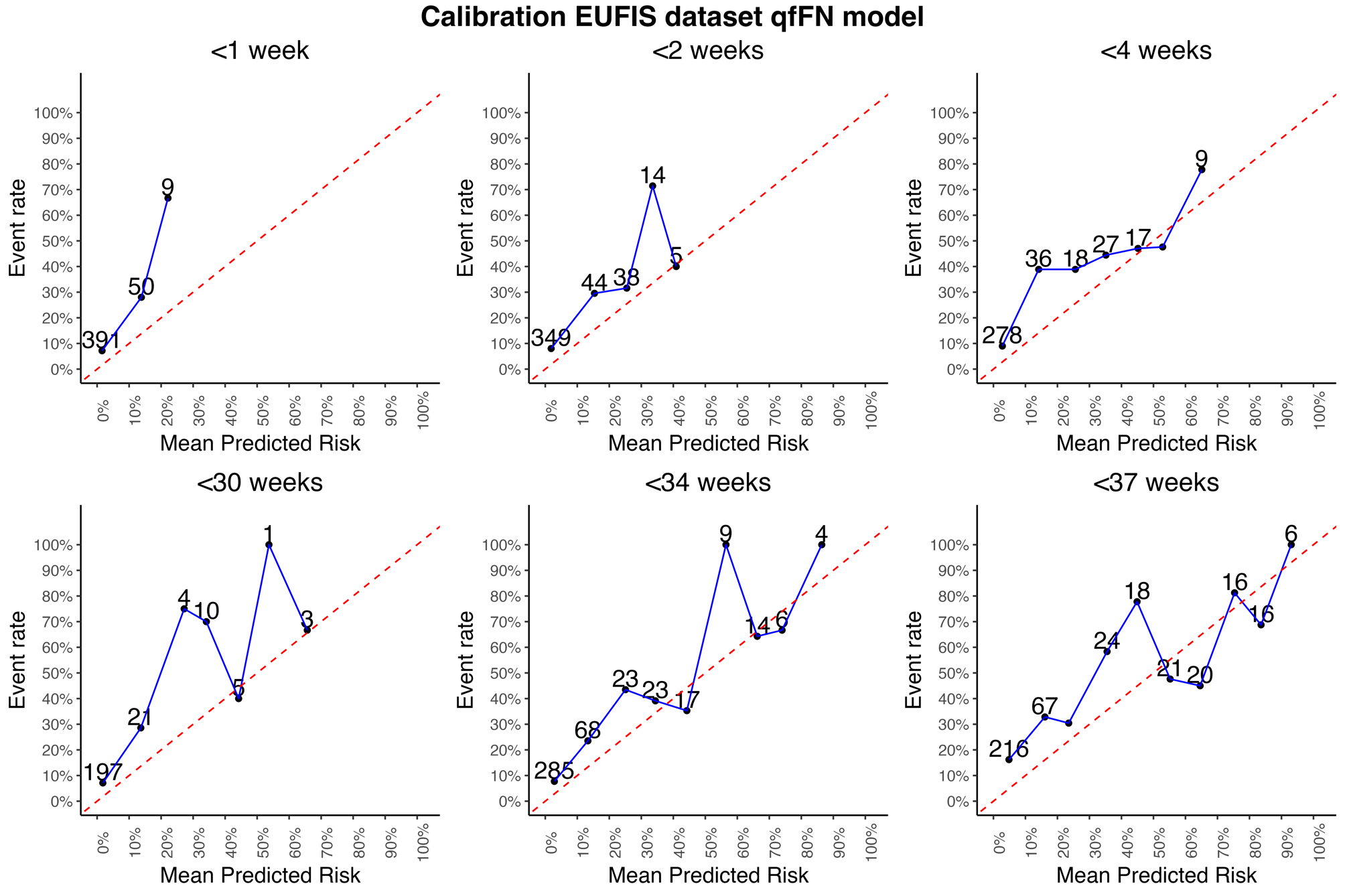


Figure S9: Calibration plots for prediction by QUiPP App v.2 of risk of spontaneous preterm birth at six predefined timepoints using quantitative fetal fibronectin in European Fibronectin Study dataset.


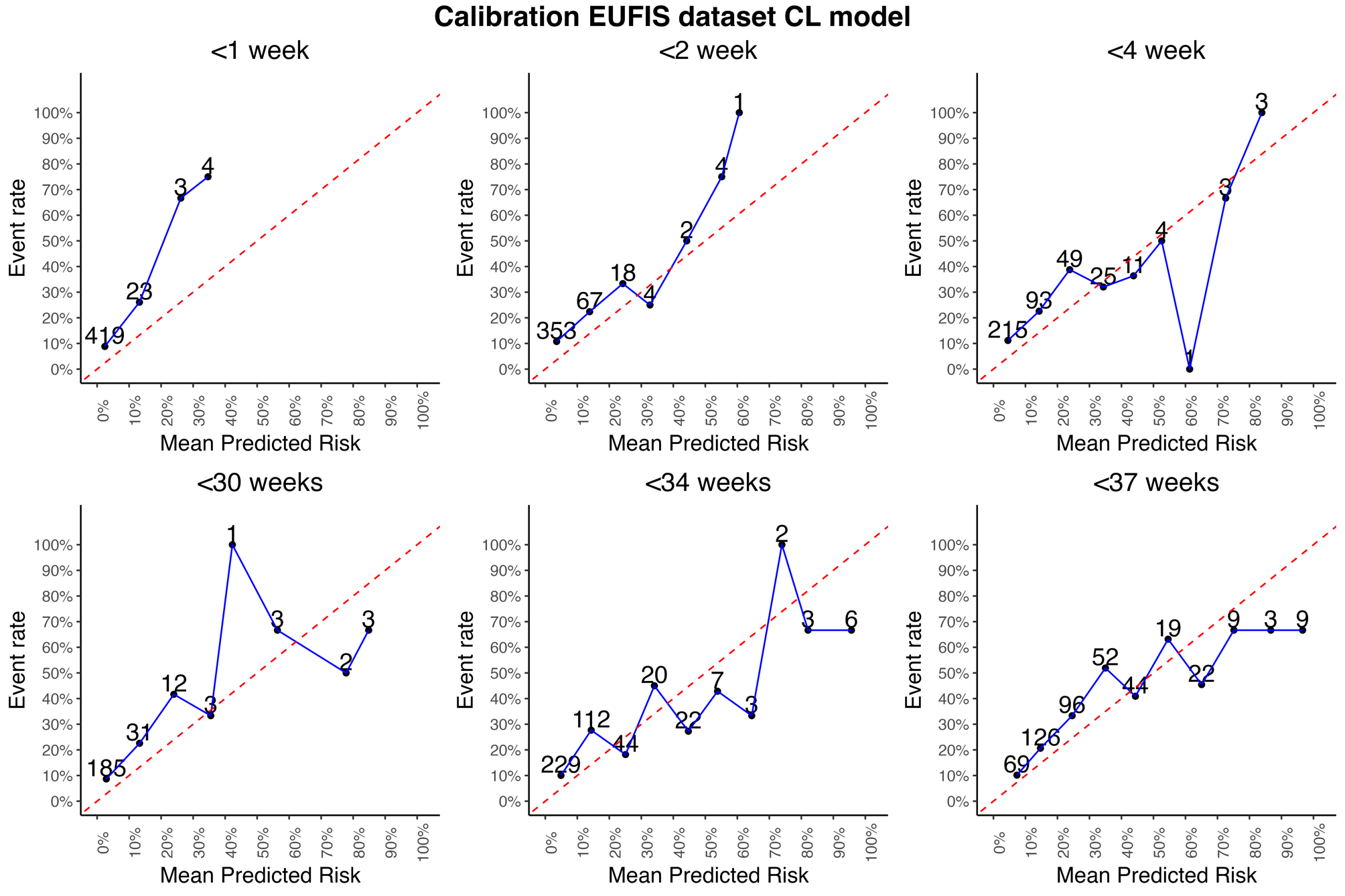


Figure S10: Calibration plots for prediction by QUiPP App v.2 of risk of spontaneous preterm birth at six predefined timepoints using cervical length in European Fibronectin Study dataset.


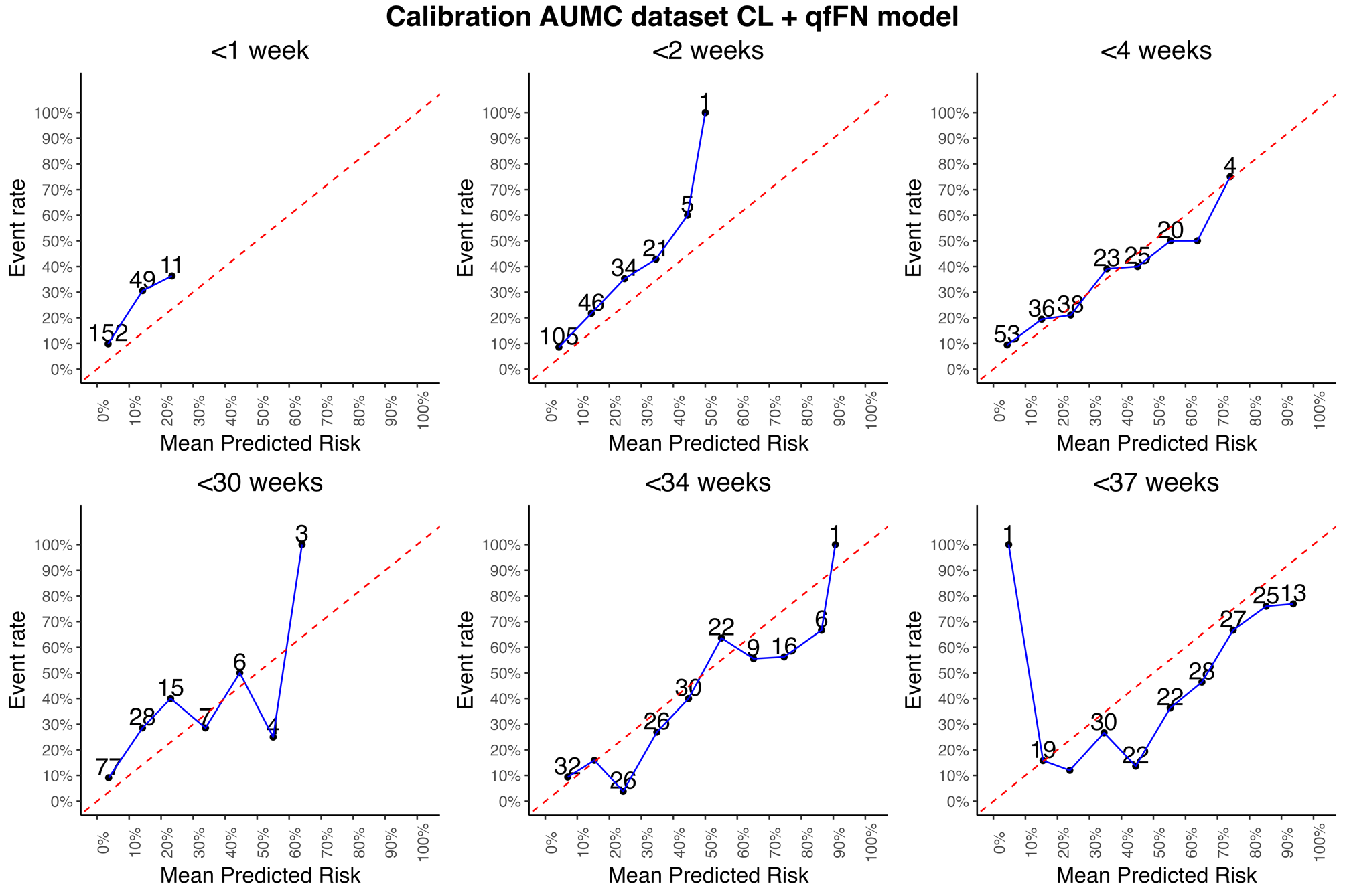


Figure S11: Calibration plots for prediction by QUiPP App v.2 of risk of spontaneous preterm birth at six predefined timepoints using cervical length plus quantitative fetal fibronectin in Amsterdam University Medical Centre dataset.


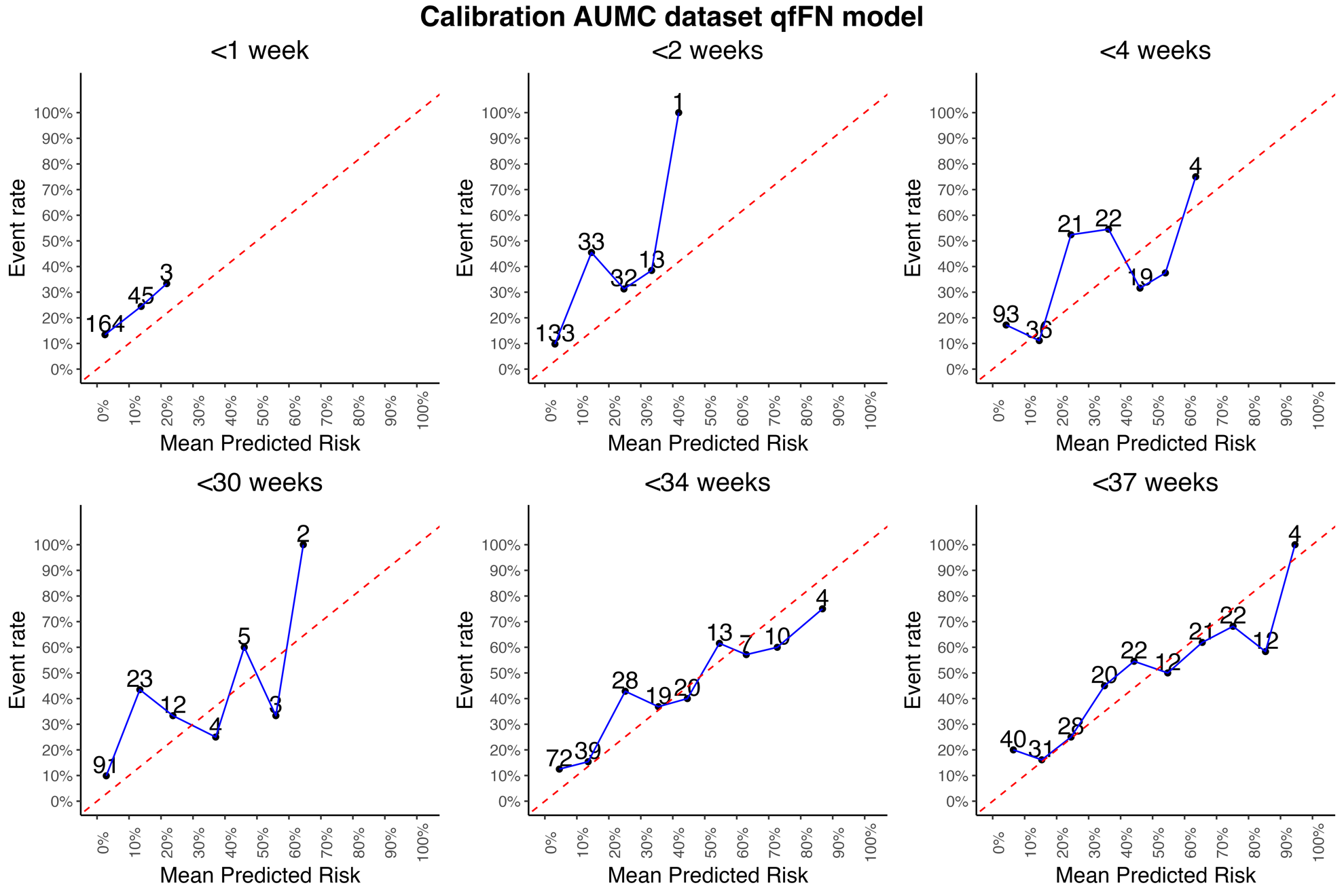


Figure S12: Calibration plots for prediction by QUiPP App v.2 of risk of spontaneous preterm birth at six predefined timepoints using quantitative fetal fibronectin in Amsterdam University Medical Centre dataset


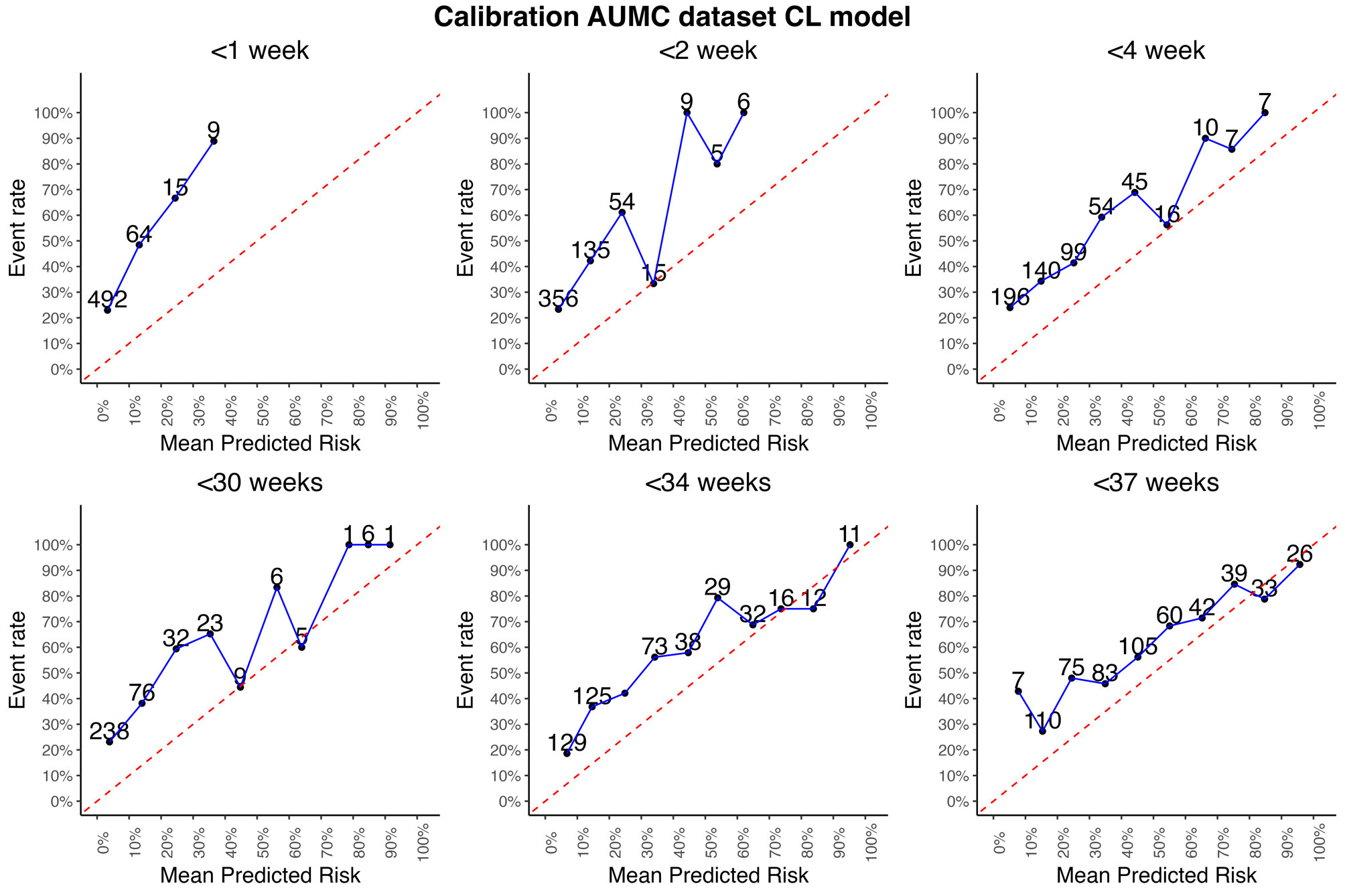


Figure S13: Calibration plots for prediction by QUiPP App v.2 of risk of spontaneous preterm birth at six predefined timepoints using cervical length in Amsterdam University Medical Centre dataset.
