# Supplementary material for: External validation of QUiPP App in three independent European cohorts of symptomatic women
Source: Ultrasound Obstet Gynecol. 2025 Jun 26;66(2):163–74. doi: 10.1002/uog.29263 (PMC12317302; doi:10.1002/uog.29263)
Supplement: Supplementary file 3 — Table S1 Inclusion and exclusion criteria for European Fibronectin Study (EUFIS) cohort, Amsterdam University Medical Center (AUMC) cohort, Ghent University Hospital (UZG) cohort and original QUiPP study Table S2 Positive and negative likelihood ratios for prediction by QUiPP App v.2 of risk of spontaneous preterm birth at six predefined timepoints using cervical length plus quantitative fetal fibronectin model in European Fibronectin Study dataset Tables S3–S5 Discrimination and calibration of QUiPP App v.2 for prediction of spontaneous preterm birth at six predefined timepoints using cervical length (CL) plus quantitative fetal fibronectin (qfFN) model (Table S3), qfFN‐only model (Table S4) and CL‐only model (Table S5) in European Fibronectin Study dataset Table S6 Positive and negative likelihood ratios for prediction by QUiPP App v.2 of risk of spontaneous preterm birth at six predefined timepoints using cervical length plus quantitative fetal fibronectin model in Amsterdam University Medical Center dataset Tables S7–S9 Discrimination and calibration of QUiPP App v.2 for prediction of spontaneous preterm birth at six predefined timepoints using cervical length (CL) plus quantitative fetal fibronectin (qfFN) model (Table S7), qfFN‐only model (Table S8) and CL‐only model (Table S9) in Amsterdam University Medical Center dataset [file UOG-66-163-s003.docx]

# Supplementary tables

Table S1: Inclusion and exclusion criteria for European Fibronectin Study (EUFIS) cohort, Amsterdam University Medical Centre (AUMC) cohort, Ghent University Hospital (UZG) cohort and original QUiPP study

| **EUFIS** | | |
| --- | --- | --- |
| **Inclusion** | **Exclusion** | |
| 24^+0^ – 34^+6^ weeks GA | PPROM | |
| Symptoms of TPTL | Prior treatment with tocolysis was initiated for longer than > 18 hours | |
| qfFN result and/or CL measurements | Established labor | |
| ≥ 18 years | Triplets or higher | |
|  | Contraindications for tocolysis:   - Lethal congenital abnormality - Suspected intrauterine infection - Nonreassuring fetal status - Placental abruption - Severe vaginal blood loss | |
|  | Cervical dilatation of > 3 cm | |
|  | | |
| **Amsterdam UMC** | | |
| **Inclusion** | **Exclusion** | |
| 23^+0^ – 34^+6^ weeks GA | PPROM | |
| Symptoms of threatened PTB | Major blood loss | |
| qfFN test result and/or CL measurement known | Established labor | |
| ≥ 18 years | Triplets or higher | |
|  | Major congenital abnormalities | |
|  | Placental abruption | |
|  | Maternal distress | |
|  | Fetal distress | |
|  | | |
| **UZ Gent** | | |
| **Inclusion** | **Exclusion** | |
| 2012-mid 2017: delivery due to sPTL between 24^+0^ – 34^+0^ weeks GA  mid 2017-2023: admission for sPTL between 24^+0^ – 34^+0^ weeks GA  Symptoms of threatened sPTB | Established labor  PPROM  Triplets or higher  Major congenital abnormalities | |
| **Original QUiPP app^13^** | | |
| **Inclusion** | | **Exclusion** |
| 23^+0^ – 34^+6^ weeks GA | | Established labor |
| Symptoms of TPTL, e.g. abdominal pain or tightening | | PPROM |
|  | | Antepartum hemorrhage |
|  | | Women with incomplete data |
|  | | Invalid or missing test results |
|  | | Sexual intercourse within the past 24 h |
|  | | Major fetal abnormality |
|  | | Triplets or higher |

*Table S2: Positive and negative likelihood ratios for prediction by QUiPP App v.2 of risk of spontaneous preterm birth at six predefined timepoints using cervical length plus quantitative fetal fibronectin model in European Fibronectin Study dataset*

| **Outcome** | **LR+ (95% CI)** | | | **LR- (95% CI)** | | |
| --- | --- | --- | --- | --- | --- | --- |
|  | 5% | 10% | 15% | 5% | 10% | 15% |
| sPTB < 1 week | 2.902 (2.292-3.622) | 3.486 (2.394-4.894) | 5.455 (3.351-8.748) | 0.312 (0.159-0.485) | 0.543 (0.374-0.712) | 0.612 (0.455-0.764) |
| sPTB < 2 weeks | 2.004 (1.747-2.294) | 2.796 (2.233-3.457) | 3.476 (2.618-4.594) | 0.169 (0.055-0.318) | 0.337 (0.199-0.491) | 0.435 (0.294-0.584) |
| sPTB < 4 weeks | 1.574 (1.425-1.732) | 1.953 (1.709-2.238) | 2.499 (2.089-3.003) | 0.093 (0.002-0.226) | 0.159 (0.061-0.290) | 0.236 (0.126-0.367) |
| sPTB < 30 weeks | 2.831 (2.329-3.476) | 4.908 (3.679-6.841) | 6.842 (4.524-10.861) | 0.043 (0.000-0.158) | 0.105 (0.005-0.242) | 0.258 (0.116-0.430) |
| sPTB < 34 weeks | 1.470 (1.349-1.612) | 1.804 (1.565-2.072) | 2.310 (1.936-2.750) | 0.128 (0.030-0.283) | 0.277 (0.150-0.438) | 0.294 (0.176-0.434) |
| sPTB < 37 weeks | 1.076 (1.035-1.119) | 1.228 (1.148-1.318) | 1.364 (1.240-1.501) | 0.165 (0.000-0.512) | 0.187 (0.060-0.377) | 0.268 (0.138-0.435) |

Table S3: Discrimination and calibration of QUiPP App v.2 for risk of spontaneous preterm birth at six predefined timepoints using cervical length plus quantitative fetal fibronectin in European Fibronectin Study dataset

| **Outcome** | **Screened** | **Events** | **Discrimination** | **Sensitivity** | | | **Calibration** | |
| --- | --- | --- | --- | --- | --- | --- | --- | --- |
|  | **n** | **%** | **AUC (95% CI)** | **% (95% CI)** | | | **Intercept** | **Slope** |
|  | | | | 5% | 10% | 15% |  | |
| sPTB < 2 weeks | 447 | 65 (14.5) | 0.82 (0.77-0.87) | 90.8 (83.1-96.9) | 75.4 (64.6-86.2) | 64.6 (52.3-76.9) | 0.25 | 0.88 |
| sPTB < 4 weeks | 447 | 95 (21.3) | 0.82 (0.77-0.87) | 96.4 (91.6-100.0) | 91.6 (85.5-97.6) | 84.3 (75.9-91.6) | -0.13 | 0.82 |
| sPTB < 30 weeks | 239 | 35 (14.6) | 0.91 (0.86-0.95) | 97.1 (91.4-100) | 91.4 (80-100) | 77.1 (62.9-91.4) | 0.84 | 1.17 |
| sPTB < 34 weeks | 446 | 89 (20.0) | 0.82 (0.76-0.87) | 94.4 (88.8-98.9) | 85.4 (77.5-92.1) | 80.9 (73.0-88.8) | -0.29 | 0.84 |
| sPTB < 37 weeks | 447 | 146 (32.7) | 0.77 (0.72-0.82) | 98.6 (96.6-100.0) | 95.9 (91.8-98.6) | 91.1 (86.3-95.2) | -0.39 | 0.71 |

Table S4: Discrimination and calibration of QUiPP App v.2 for risk of spontaneous preterm birth at six predefined timepoints using quantitative fetal fibronectin in European Fibronectin Study dataset

| **Outcome** | **Screened** | **Events (%)** | **Discrimination** |  | | | **Calibration** | |
| --- | --- | --- | --- | --- | --- | --- | --- | --- |
|  |  |  | **AUC (95% CI)** | **Sensitivity % (95% CI)** | | | **Intercept** | **Slope** |
|  | | | | 5% | 10% | 15% |  | |
| sPTB < 2 weeks | 450 | 65 (14.4) | 0.81 (0.75-0.86) | 70.8 (60.0-81.5) | 56.9 (44.6-69.2) | 49.2 (36.9-61.5) | 0.38 | 0.68 |
| sPTB < 4 weeks | 450 | 95 (21.1) | 0.80 (0.75-0.85) | 80.7 (72.3-89.2) | 69.9 (60.2-79.5) | 60.2 (49.4-69.9) | 0.23 | 0.66 |
| sPTB < 30 weeks | 241 | 35 (14.5) | 0.89 (0.84-0.94) | 85.7 (74.3-97.1) | 60 (42.9-74.3) | 51.4 (34.3-68.6) | 1.12 | 0.93 |
| sPTB < 34 weeks | 449 | 89 (19.8) | 0.81 (0.76-0.86) | 83.5 (78.6-88.3) | 75.3 (66.3-84.3) | 64.0 (53.9-74.2) | 0.28 | 0.76 |
| sPTB < 37 weeks | 450 | 148 (32.9) | 0.75 (0.70-0.80) | 87.8 (82.4-92.6) | 76.4 (69.6-83.1) | 73.0 (65.5-79.7) | 0.22 | 0.62 |

Table S5: Discrimination and calibration of QUiPP App v.2 for risk of spontaneous preterm birth at six predefined timepoints using cervical length in European Fibronectin Study dataset

| **Outcome** | **Screened** | **Events (%)** | **Discrimination** |  | | | **Calibration** | |
| --- | --- | --- | --- | --- | --- | --- | --- | --- |
|  |  |  | **AUC (95% CI)** | **Sensitivity % (95% CI)** | | | **Intercept** | **Slope** |
|  | | | | 5% | 10% | 15% |  | |
| sPTB < 2 weeks | 449 | 65 (14.5) | 0.71 (0.65-0.78) | 67.7 (56.9-78.5) | 41.5 (29.2-53.8) | 29.2 (18.4-41.5) | 0.13 | 0.66 |
| sPTB < 4 weeks | 449 | 95 (21.2) | 0.72 (0.66-0.78) | 89.2 (81.9-95.2) | 71.1 (60.2-80.7) | 57.8 (47.0-68.7) | 0.07 | 0.70 |
| sPTB < 30 weeks | 240 | 35 (14.6) | 0.79 (0.72-0.86) | 71.4 (57.1-85.7) | 54.3 (37.1-71.4) | 37.1 (20-54.3) | 0.22 | 0.71 |
| sPTB < 34 weeks | 448 | 89 (19.9) | 0.72 (0.67-0.78) | 93.3 (87.6-97.8) | 74.2 (65.2-83.1) | 57.3 (47.2-67.4) | -0.27 | 0.60 |
| sPTB < 37 weeks | 449 | 146 (32.5) | 0.71 (0.66-0.76) | 98.6 (96.6-100.0) | 95.2 (91.1-97.9) | 85.6 (79.5-91.1) | -0.11 | 0.62 |

Table S6: Positive and negative likelihood ratios for prediction by QUiPP App v.2 of risk of spontaneous preterm birth at six predefined timepoints using cervical length plus quantitative fetal fibronectin model in Amsterdam University Medical Centre dataset

| **Outcome** | **LR+ (95% CI)** | | | **LR- (95% CI)** | | |
| --- | --- | --- | --- | --- | --- | --- |
|  | 5% | 10% | 15% | 5% | 10% | 15% |
| sPTB < 1 week | 1.940 (1.502-2.463) | 2.529 (1.610-3.669) | 3.272 (1.534-6.457) | 0.340 (0.136-0.601) | 0.554 (0.342-0.776) | 0.759 (0.573-0.923) |
| sPTB < 2 weeks | 1.409 (1.209-1.621) | 1.882 (1.483-2.367) | 2.272 (1.639-3.120) | 0.252 (0.057-0.548) | 0.348 (0.159-0.583) | 0.472 (0.280-0.686) |
| sPTB < 4 weeks | 1.183 (1.067-1.302) | 1.338 (1.167-1.529) | 1.387 (1.160-1.651) | 0.258 (0.004-0.651) | 0.268 (0.070-0.564) | 0.422 (0.196-0.711) |
| sPTB < 30 weeks | 1.616 (1.273-2.012) | 2.108 (1.514-2.904) | 2.226 (1.269-3.480) | 0.288 (0.063-0.607) | 0.367 (0.148-0.636) | 0.581 (0.345-0.840) |
| sPTB < 34 weeks | - | 1.183 (2.067-1.297) | 1.342 (1.184-1.521) | - | 0.245 (0.004-0.631) | 0.210 (0.047-0.469) |
| sPTB < 37 weeks | - | - | - | - | - | - |

Table S7: Discrimination and calibration of QUiPP App v.2 for risk of spontaneous preterm birth at six predefined timepoints using cervical length plus quantitative fetal fibronectin in Amsterdam University Medical Centre dataset

| **Outcome** | **Screened** | **Events** | **Discrimination** | **Sensitivity** | | | **Calibration** | |
| --- | --- | --- | --- | --- | --- | --- | --- | --- |
|  | **n** | **n (%)** | **AUC (95% CI)** | **% (95% CI)** | | | **Intercept** | **Slope** |
|  | | | | 5% | 10% | 15% |  | |
| sPTB < 2 weeks | 212 | 44 (20.8) | 0.76 (0.69-0.84) | 90.9 (81.8-97.7) | 79.5 (65.9-90.0) | 65.9 (52.3-79.5) | 0.41 | 0.91 |
| sPTB < 4 weeks | 212 | 58 (27.4) | 0.71 (0.63-0.79) | 94.8 (87.9-100.0) | 91.4 (84.4-98.3) | 82.8 (72.4-91.4) | -0.29 | 0.62 |
| sPTB < 30 weeks | 140 | 30 (21.4) | 0.76 (0.67-0.85) | 86.7  (73.3-96.7) | 76.7  (60-90) | 56.7  (40-73.3) | 0.22 | 0.73 |
| sPTB < 34 weeks | 212 | 63 (29.7) | 0.76 (0.68-0.83) | 98.4  (95.2-100) | 95.2  (88.9-100) | 93.7  (87.3-98.4) | -0.37 | 0.83 |
| sPTB < 37 weeks | 212 | 86 (40.6) | 0.76 (0.69-0.83) | 98.8  (96.5-100) | 98.8  (96.5-100) | 95.3  (90.7-98.8) | -0.62 | 0.81 |

Table S8: Discrimination and calibration of QUiPP App v.2 for risk of spontaneous preterm birth at six predefined timepoints using quantitative fetal fibronectin in Amsterdam University Medical Centre dataset

| **Outcome** | **Screened** | **Events (%)** | **Discrimination** |  | | | **Calibration** | |
| --- | --- | --- | --- | --- | --- | --- | --- | --- |
|  |  |  | **AUC (95% CI)** | **Sensitivity % (95% CI)** | | | **Intercept** | **Slope** |
|  | | | | 5% | 10% | 15% |  | |
| sPTB < 2 weeks | 212 | 44 (20.8) | 0.73 (0.66-0.81) | 79.5 (65.9-90.9) | 70.5 (56.8-84.1) | 52.3 (36.4-65.9) | 0.27 | 0.64 |
| sPTB < 4 weeks | 212 | 58 (27.4) | 0.69 (0.62-0.77) | 91.4 (84.5-98.3) | 72.4 (60.3-84.5) | 67.2 (55.2-79.3) | -0.12 | 0.49 |
| sPTB < 30 weeks | 140 | 30 (21.4) | 0.76 (0.67-0.86) | 86.7 (73.3-96.7) | 70 (53.3-86.7) | 43.3 (26.7-60.0) | 0.29 | 0.61 |
| sPTB < 34 weeks | 212 | 63 (29.7) | 0.74 (0.66-0.81) | 92.1 (84.1-98.4) | 85.7 (76.2-93.7) | 81.0 (71.4-90.5) | -0.05 | 0.63 |
| sPTB < 37 weeks | 212 | 86 (40.6) | 0.73 (0.67-0.80) | 96.5 (91.8-100.0) | 90.7 (83.7-96.5) | 88.4 (81.4-94.2) | -0.04 | 0.62 |

Table S9: Discrimination and calibration of QUiPP App v.2 for risk of spontaneous preterm birth at six predefined timepoints using cervical length in Amsterdam University Medical Centre dataset

| **Outcome** | **Screened** | **Events (%)** | **Discrimination** |  | | | **Calibration** | |
| --- | --- | --- | --- | --- | --- | --- | --- | --- |
|  |  |  | **AUC (95% CI)** | **Sensitivity % (95% CI)** | | | **Intercept** | **Slope** |
|  | | | | 5% | 10% | 15% |  | |
| sPTB < 2 weeks | 580 | 197 (34.0) | 0.70 (0.66-0.75) | 77.2 (71.1-82.7) | 57.9 (50.8-65.0) | 39.6 (33.0-46.7) | 0.95 | 0.64 |
| sPTB < 4 weeks | 580 | 234 (40.3) | 0.70 (0.66-0.75) | 90.4 (86.9-93.9) | 79.6 (74.3-84.8) | 70.4 (64.3-76.1) | 0.59 | 0.62 |
| sPTB < 30 weeks | 397 | 138 (34.8) | 0.72 (0.66-0.77) | 75.4 (68.1-82.6) | 60.1 (51.2-68.1) | 47.8 (39.9-56.5) | 0.77 | 0.59 |
| sPTB < 34 weeks | 579 | 258 (44.6) | 0.72 (0.67-0.76) | 98.4 (96.9-99.6) | 90.7 (86.8-94.2) | 78.7 (73.6-83.7) | 0.57 | 0.70 |
| sPTB < 37 weeks | 580 | 320 (55.2) | 0.71 (0.67-0.76) | 100.0 (100.0-100.0) | 99.1 (97.8-100) | 94.4 (91.9-96.9) | 0.45 | 0.72 |
